# Identification of KasA as the cellular target of an anti-tubercular scaffold

**DOI:** 10.1038/ncomms12581

**Published:** 2016-09-01

**Authors:** Katherine A. Abrahams, Chun-wa Chung, Sonja Ghidelli-Disse, Joaquín Rullas, María José Rebollo-López, Sudagar S. Gurcha, Jonathan A. G. Cox, Alfonso Mendoza, Elena Jiménez-Navarro, María Santos Martínez-Martínez, Margarete Neu, Anthony Shillings, Paul Homes, Argyrides Argyrou, Ruth Casanueva, Nicholas J. Loman, Patrick J. Moynihan, Joël Lelièvre, Carolyn Selenski, Matthew Axtman, Laurent Kremer, Marcus Bantscheff, Iñigo Angulo-Barturen, Mónica Cacho Izquierdo, Nicholas C. Cammack, Gerard Drewes, Lluis Ballell, David Barros, Gurdyal S. Besra, Robert H. Bates

**Affiliations:** 1 Institute of Microbiology and Infection, School of Biosciences, University of Birmingham, Edgbaston, Birmingham B15 2TT, UK; 2 GlaxoSmithKline, Gunnels Wood Road, Stevenage SG1 2NY, UK; 3 Cellzome—a GSK Company, Meyerhofstrasse 1, 69117 Heidelberg, Germany; 4 Diseases of the Developing World, GlaxoSmithKline, Severo Ochoa 2, Tres Cantos, 28760 Madrid, Spain; 5 GlaxoSmithKline, 709 Swedeland Road, PO Box 1539, King of Prussia, Pennsylvania 19406-0939, USA; 6 Centre National de la Recherche Scientifique FRE 3689, Centre d'études d'agents Pathogènes et Biotechnologies pour la Santé, Université de Montpellier, 1919 route de Mende, 34293 Montpellier, France; 7 INSERM, CPBS, 34293 Montpellier, France

## Abstract

Phenotypic screens for bactericidal compounds are starting to yield promising hits against tuberculosis. In this regard, whole-genome sequencing of spontaneous resistant mutants generated against an indazole sulfonamide (GSK3011724A) identifies several specific single-nucleotide polymorphisms in the essential *Mycobacterium tuberculosis* β-ketoacyl synthase (*kas*) A gene. Here, this genomic-based target assignment is confirmed by biochemical assays, chemical proteomics and structural resolution of a KasA-GSK3011724A complex by X-ray crystallography. Finally, *M. tuberculosis* GSK3011724A-resistant mutants increase the *in vitro* minimum inhibitory concentration and the *in vivo* 99% effective dose in mice, establishing *in vitro* and *in vivo* target engagement. Surprisingly, the lack of target engagement of the related β-ketoacyl synthases (FabH and KasB) suggests a different mode of inhibition when compared with other Kas inhibitors of fatty acid biosynthesis in bacteria. These results clearly identify KasA as the biological target of GSK3011724A and validate this enzyme for further drug discovery efforts against tuberculosis.

## Abstract (web summary)

Screens for bactericidal compounds have resulted in promising anti-tubercular hits. Here, the authors analyse in detail the target of an indazole sulfonamide (GSK3011724A), and find that it has a different mode of inhibition compared to other Kas inhibitors of fatty acid biosynthesis in bacteria.

## Graphical abstract

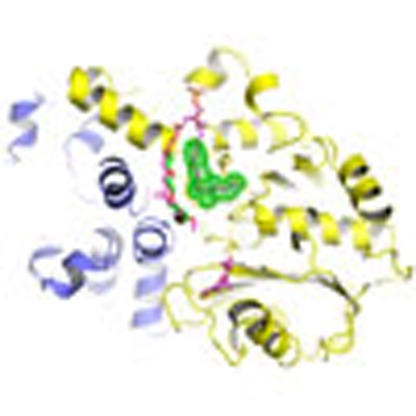

After many years of relatively little attention, *Mycobacterium tuberculosis*, the causative agent of tuberculosis (TB), has re-emerged as a priority in the area of neglected diseases^1^. The standard 6-month treatment for TB has remained essentially unchanged over several decades^2^. Unfortunately, poor patient compliance and other factors have led to an increasing prevalence of drug resistance. In 2013, the World Health Organization (WHO) recorded ∼480,000 new cases of multi-drug-resistant (MDR)-TB, resistant to both the front-line drugs, isoniazid (INH) and rifampicin (RIF)^3^. Without these two front-line drugs, MDR-TB generally requires 24 months of treatment with a variety of second-line antibiotics, which are poorly tolerated. Due to this continued spread of MDR and extensively-drug-resistant forms of *M. tuberculosis*, the WHO has declared TB as a world health emergency^3^. Therefore, new drugs that can complement existing front-line treatment regimens are urgently required.

In 2013 and updated in 2015, GlaxoSmithKline published a set of 228 small molecule hits from two phenotypic screening campaigns against *M. tuberculosis*^4^,^5^. Of those 228 compounds, a number have been explored via medicinal chemistry for potential optimization to drug leads and clinical candidates. Phenotypic screening also offers the opportunity to identify novel biological targets by molecular mode of action (MoA) studies, which is generally achieved through a variety of omics-based technologies^6^,^7^,^8^,^9^,^10^. GSK3011724A is a small molecule inhibitor from the aforementioned 228 phenotypic screening hits^4^,^5^. In this study, we provide an initial profiling of GSK3011724A as a suitable starting point for ‘hit' to ‘lead' drug development based on its *in vitro* and *in vivo* characteristics. In addition, through MoA studies, we identify the *M. tuberculosis* cellular target of GSK3011724A to be KasA, a β-ketoacyl synthase. Target assignment reveals specificity of GSK3011724A for KasA with a binding site distinct from other known Kas inhibitors. This different MoA creates new potential for this recognized target in future TB drug discovery efforts.

## Results

### *In vitro* profiling of GSK3011724A

**Figure 1:**
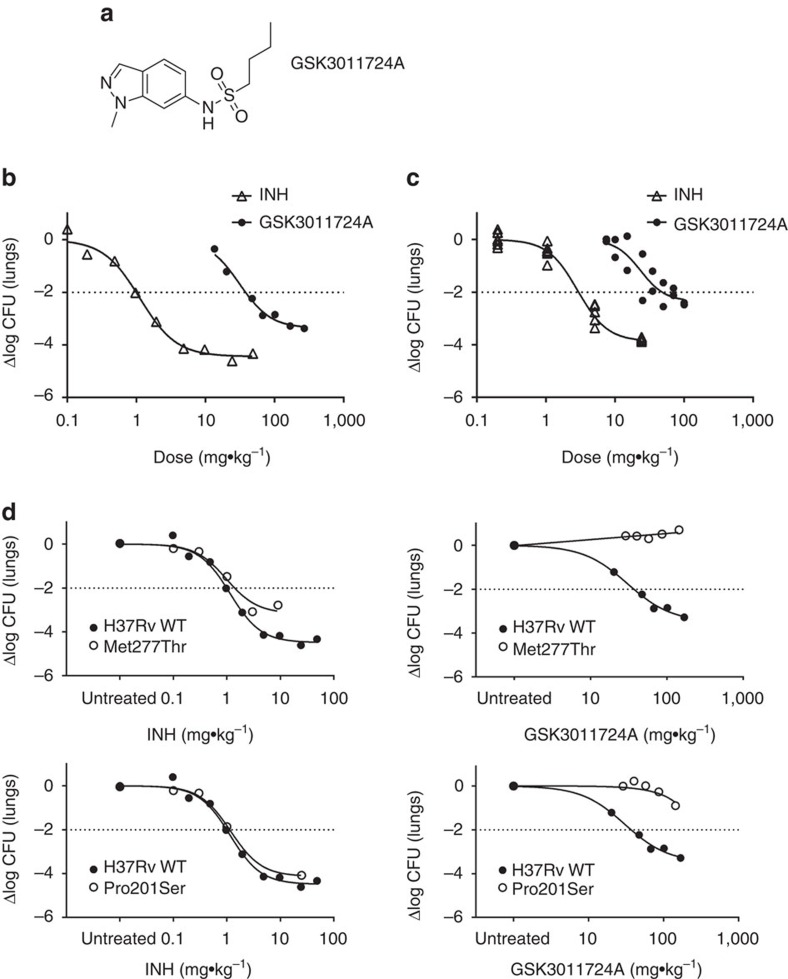
The anti-tubercular activity of GSK3011724A. (**a**) Chemical structure of GSK3011724A. (**b**,**c**) GSK3011724A was evaluated in an acute (**b**) and chronic (**c**) model of murine TB infection. Each symbol represents data from a single mouse. A two-fold log c.f.u. reduction equates to a 2 log c.f.u. difference compared with untreated mice at the end of the treatment period. Untreated mice gave a log c.f.u. count of 7.1±0.1 (mean±s.d., *n*=5 mice) in the acute assay and 5.8±0.26 (mean±s.d., *n*=7 mice for the INH experiment) or 6.0±0.5 (mean±s.d., *n*=5 mice for GSK3011724A experiment) in the chronic assay. (**d**) Comparison of the *in vivo* efficacy of GSK3011724A and INH against *M. tuberculosis* H37Rv wild-type (H37Rv WT) and *M. tuberculosis* KasA mutant strains. Mice were infected with each strain and administered with INH or GSK3011724A at different doses during the acute phase (days 1 to 8 after infection). Each dot represents data from a single mouse. Log c.f.u. counts are shown as the difference with respect to the untreated group infected with each strain (Δlog c.f.u. for each mouse).

From the outset, GSK3011724A, an indazole sulfonamide (Fig. 1a), represented an attractive compound for early stage drug discovery based on its anti-mycobacterial potency, small size and moderate lipophilicity (Table 1). Further profiling *in vitro* using standard assays, such as the hERG ion channel and cytochrome P450 isoforms supported this view (Table 1). Sensitivity testing of 18 Gram-positive and Gram-negative bacterial species with GSK3011724A demonstrated selectivity of this compound for *M. tuberculosis*. In addition, GSK3011724A showed negligible activity against a panel of unrelated proteins (Supplementary Table 1). These desirable characteristics demonstrated the clear potential of GSK3011724A as a starting point for medicinal chemistry optimization. A description of this optimization effort along with a thorough discussion of the drug development potential and liabilities of this series will be the subject of a future publication.

### *In vivo* profiling of GSK3011724A

GSK3011724A was progressed into pharmacokinetic (PK) and efficacy experiments to generate an *in vivo* profile. Although doses above 400 mg kg^−1^ were not well tolerated by mice, multiple days of dosing up to 300 mg kg^−1^ once daily proceeded with no weight loss or other adverse effects. The *in vivo* PK of GSK3011724A in mice was disproportional based on dosing. At low doses, clearance close to the liver blood flow rate was observed (in agreement with the *in vitro* clearance (Cli) in mouse microsomes), but as the doses were increased, the maximum concentration (Cmax) and area under the curve values grew disproportionally (Table 2). This observation suggests a saturation of the mechanism of clearance, and may help to explain the reduction in tolerability between 300 and 400 mg kg^−1^ dosing.

The first stage of efficacy testing involved an acute model of infection^11^. This murine model involved infecting mice with a high inoculum (10^5^ colony-forming units (c.f.u.)) of *M. tuberculosis* H37Rv wild type and beginning treatment the next day. GSK3011724A (and INH as a positive control) were administered once daily for 8 days, while the bacilli were in the exponential growth phase. Despite exhibiting a bacteriostatic effect *in vitro* (Supplementary Fig. 1), GSK3011724A demonstrated a significant cidal effect in this murine model with a ∼3.5 log c.f.u. reduction relative to untreated controls at 200 mg kg^−1^ (Fig. 1b). The effects of GSK3011724A are similar to linezolid, which has been shown to be bacteriostatic *in vitro*^12^, but exhibits cidality *in vivo*^11^. Importantly, a clear dose–response was observed, with the ED_99_ (dose required to reduce bacterial load by 99%) determined as 38 mg kg^−1^. This level of ED_99_ is only moderately higher than most of the gold standard TB drugs^11^.

GSK3011724A (and INH as a positive control) was also evaluated in a chronic infection assay in which mice were infected with a lower inoculum of *M. tuberculosis* H37Rv (10^2^ c.f.u.) and left untreated for 6 weeks, allowing the bacilli to reach a steady state. GSK3011724A was then dosed daily for 2 months. The dose–response curve in this chronic assay was shifted to higher doses than in the acute model, but the targeted >2 log c.f.u. reduction was achieved at 100 mg kg^−1^ for GSK3011724A, producing a 2.4 log c.f.u. reduction (Fig. 1c). These data clearly establish GSK3011724A as an active compound in both the acute and chronic *in vivo* murine models of *M. tuberculosis* infection. Importantly, if GSK3011724A were demonstrated to inhibit an unexploited potential antimicrobial target, these data would provide a significant level of validation and confidence for further exploration of that target for TB drug discovery.

### GSK3011724A target identification

A fundamental strategy in drug discovery is establishing the MoA of inhibitory compounds. Following the identification and validation of the molecular target, target-specific optimization of the compound can be pursued to improve efficacy and reduce toxicity. Due to the recent successes of utilizing whole-genome sequencing (WGS) of spontaneous resistant mutants as a primary step in the elucidation of the target of phenotypic hits^6^,^7^,^9^, this methodology was used to establish the target of GSK3011724A.

The minimum inhibitory concentration (MIC) of GSK3011724A in *Mycobacterium bovis* Bacillus Calmette–Guérin (BCG) was determined to be 0.5 μM. Spontaneous resistant mutants were initially generated using *M. bovis* BCG at 5 × , 10 × and 20 × the MIC of GSK3011724A, with frequencies of resistance (FoR) of 12 × 10^−8^, 3 × 10^−8^ and 2 × 10^−8^, respectively. The FoR was subsequently determined against *M. tuberculosis* at 2.5 μM (10 × MIC of GSK3011724A on solid media, 0.25 μM) giving a frequency of 9.5 × 10^−7^. This FoR is slightly higher than normally desired but is lower than that of INH.

From the total of 17 *M. bovis* BCG spontaneous resistant mutants generated, eight were selected for WGS. Six of the eight *M. bovis* BCG mutants were found to possess a number of high-quality (statistically relevant) single-nucleotide polymorphisms (SNP) compared with the sequenced wild-type reference strain (Genbank accession number NC_008769.1), all of which located to the gene annotated *kasA*, encoding an essential β-ketoacyl synthase involved in mycolic acid biosynthesis^13^ (Table 3). The data offered the first evidence that KasA is the target of GSK3011724A, providing a starting point for validation studies. Subsequently, instead of using WGS, the *kasA* gene from 12 isolated *M. tuberculosis* resistant mutants was specifically sequenced following amplification by PCR. The data confirmed the results from *M. bovis* BCG, with eleven of the twelve mutants showing SNPs in *kasA* (Table 3). The WGS results of the two remaining *M. bovis* BCG mutants did not reveal SNPs of a high frequency; no gene contained a SNP of greater than 50% frequency, corresponding to the percentage of SNP in the cell population from which the genomic DNA was prepared.

### KasA target validation

**Figure 2:**
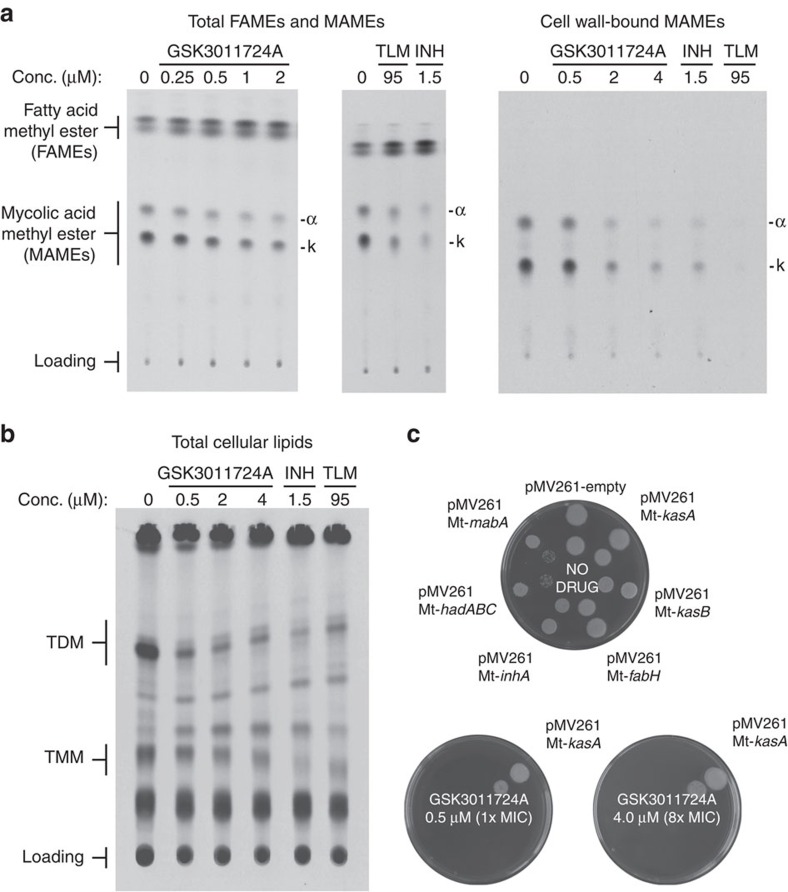
Inhibition of mycolic acid biosynthesis by GSK3011724A. (**a**) *M. bovis* BCG cultures, labelled with [^14^C]-acetate, were treated with GSK3011724A, TLM and INH. The total FAMEs and MAMEs were extracted and analysed by autoradiography-TLC using equal counts (25,000 c.p.m.) for each lane, respectively (left and middle panels). In addition, cell wall-bound MAMEs were isolated and an equal aliquot (5%) was analysed by autoradiography-TLC for each lane, respectively (right panel). (**b**) *M. bovis* BCG total cellular lipids containing TDM/TMM were extracted following labelling and drug treatment (GSK3011724A, TLM and INH) and analysed by autoradiography-TLC using equal counts (25,000 c.p.m.) for each lane, respectively. (**c**) Impact on the MIC of GSK3011724A upon the overexpression of members of FAS-II in *M. bovis* BCG. The overexpression constructs of *M. tuberculosis* enzymes (Mt) pMV261-Mt-*kasA*, pMV261-Mt-*kasB*, pMV261-Mt-*fabH*, pMV261-Mt-*inhA*, pMV261-Mt-*hadABC* and pMV261-Mt-*mabA* were electroporated into *M. bovis* BCG and the MIC of GSK3011724A was evaluated with reference to *M. bovis* BCG pMV261. The outer and inner spots represent 10^3^ and 10^2^ cells plated, respectively.

Following the target identification of KasA by WGS, a variety of techniques were utilized to confirm its role as the biological target of GSK3011724A. Firstly, the inhibition of mycolic acid biosynthesis by GSK3011724A was investigated. *M. bovis* BCG was labelled using [^14^C]-acetate and treated with increasing concentrations of GSK3011724A, with INH and the KasA inhibitor thiolactomycin (TLM) used as positive controls. Following drug treatment, the total fatty acid methyl esters (FAMEs) and α-mycolate and keto-mycolic acid methyl esters (α- and k-MAMEs) were extracted and analysed by autoradiography thin-layer chromatography (TLC). As shown in Fig. 2a (left panel) there was a clear visual dose-dependent reduction in the production of α-MAMEs and k-MAMEs upon treatment with GSK3011724A. This characteristic profile of total MAMEs inhibition and FAMEs accumulation mirrors the known fatty acid synthase (FAS)-II inhibitors TLM and INH (Fig. 2a, middle panel). When spots were excised from the TLC and quantified by scintillation counting (Table 4), GSK3011724A at 4 × MIC in comparison to the non-treated control produced a 55.2% and 42.3% reduction in α-MAMEs and k-MAMEs, respectively. In contrast, quantification of the levels of FAMEs increased by 186.5% at 4 × MIC. When compared with the known KasA inhibitor TLM, GSK3011724A at 1 × MIC afforded a similar level of inhibition of α-MAMEs and k-MAMEs, and an increase in FAMEs synthesis, respectively (Table 4). A slightly less pronounced effect at their relative MICs was observed for INH in comparison to GSK3011724A (Table 4). The inhibition of mycolic acid biosynthesis by GSK3011724A was even more apparent when cell wall-bound α- and k-MAMEs were analysed, with an almost visual complete inhibition in a dose-dependant manner, which was similar to INH and TLM (Fig. 2a, right panel). To investigate whether GSK3011724A affects the synthesis of other mycobacterial lipids, addressing the potential of additional cell wall-associated targets, the total cellular lipids (and fractionated apolar lipids and polar phospholipids) were extracted and analysed following drug treatment. Firstly, a decrease in trehalose dimycolate (TDM), trehalose monomycolate (TMM) and glycerol monomycolate was observed with GSK3011724A (and TLM and INH), consistent with inhibition of mycolic acid biosynthesis (Fig. 2b; Table 4 and Supplementary Fig. 2a). Secondly, no significant differences were observed in lipid composition when apolar lipids and polar phospholipids were analysed by autoradiography-TLC (Supplementary Fig. 2a,b), suggesting that GSK3011724A does not target other lipid biosynthetic pathways, and is specific for KasA.

To further corroborate that GSK3011724A inhibits mycolic acid biosynthesis and, more specifically, targets KasA, the impact on the MIC was investigated using strains of *M. bovis* BCG overexpressing components of FAS-II. The mycobacterial expression vectors, pMV261 containing *M. tuberculosis kasA*^14^, *kasB*^14^, *fabH*^15^, *inhA*^16^, *hadABC*^17^ and *mabA*^16^, were electroporated into *M. bovis* BCG and growth analysed at a concentration range with respect to the MIC of GSK3011724A (Fig. 2c). *M. bovis* BCG containing the pMV261 empty vector exhibited no change in sensitivity to GSK3011724A compared with the wild-type untransformed strain, with an MIC of 0.5 μM (Fig. 2c). This was also apparent for the pMV261 constructs containing *kasB*, *inhA*, *fabH*, *hadABC* and *mabA* (Fig. 2c). However, ample growth of the KasA overexpressor strain was observed at 4 μM, indicating an increase in resistance, and a MIC shift of >8 × (Fig. 2c), providing further evidence to support KasA as the cellular target of GSK3011724A.

Previous studies have reported KasA activity in an *in vitro* condensing assay using purified recombinant proteins^18^. This assay was replicated to demonstrate the specific inhibition of KasA by GSK3011724A. Initially, FabD was used to generate [^14^C]-malonyl-AcpM from holo-AcpM and [^14^C]-malonyl-CoA. KasA, in the absence or presence of GSK3011724A, elongated C_16_-AcpM in a condensation reaction with [^14^C]-malonyl-AcpM. Treatment with 1 μM of GSK3011724A provided a 58.5% inhibition of KasA activity (based on triplicate data, Supplementary Fig. 3).

**Figure 3:**
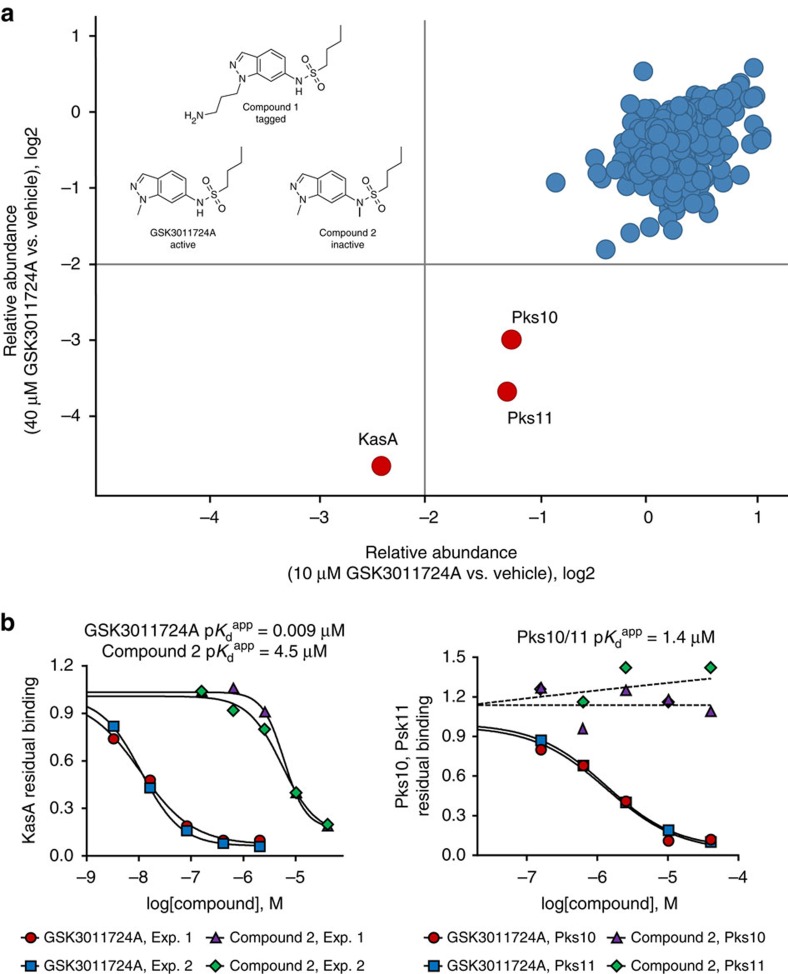
Chemoproteomics profiling of GSK3011724A. (**a**) A propylamine-tagged derivative of GSK3011724A (**1**, inset) was synthesized and covalently immobilized to NHS-activated sepharose. Beads were incubated with *M. bovis* BCG extract either in the presence of vehicle (DMSO) or GSK3011724A (10 μM, 40 μM). Proteins captured by the beads in both conditions were quantified by LC–MS/MS analysis. KasA, Pks10 and Pks11 were identified as potential targets of GSK3011724A by virtue of their reduced capturing in the presence of excess GSK3011724A. (**b**) Generation of IC_50_ values for KasA, Pks10 and Pks11. The chemoproteomic experiment was performed as in **a** but over a range of concentrations of the competing ‘free' inhibitor GSK3011724A (2–0.003 μM for KasA, 40–0.16 μM for Pks10 and Pks11) and a structurally related inactive analogue, **2** (40–0.16 μM). Apparent dissociation constants for GSK3011724A were determined from two independent experiments.

To investigate whether GSK3011724A exerts its effect by directly binding to KasA, we employed a chemoproteomics strategy^19^,^20^. A tagged analogue (**1**) with an MIC of 49 μM was generated, which was covalently linked to Sepharose beads (Fig. 3a). The tagged analogue-(**1**)-derivatized beads were incubated with *M. bovis* BCG extracts, in the absence or presence of an excess of unbound GSK3011724A. In this competition-binding format, target proteins would be expected to bind to the beads predominantly in the absence of excess competing compound. After washing of the beads, bound proteins were digested with trypsin and subjected to quantitative mass spectrometry. Among >2,000 proteins identified, only three proteins were efficiently competed off the beads by excess GSK3011724A: KasA, and, to a lesser degree, the non-essential polyketide synthases Pks10 and Pks11 (refs 21, 22; Fig. 3a; Supplementary Data 1 and 2). The experiment was repeated in a concentration-dependent format to determine half-maximal inhibition (IC_50_) values that refer to the concentration of compound (GSK3011724A) required to competitively block 50% of the target (that is, KasA) from binding to the beads (Fig. 3b; Supplementary Data 3 and 4). These IC_50_ values are a measure of target affinity, but are also affected by the affinity of the target for the bead-immobilized ligand. The latter effect can be deduced by measuring the depletion of the target proteins (KasA, Pks10 and Pks11) by the beads. Thus, apparent dissociation constants (*K*_d_^app^) can be determined, which are largely independent from the bead ligand^19^,^23^. The *K*_d_^app^ value was determined as 9 nM for KasA, suggesting a high level of affinity for the enzyme. In excellent agreement with these chemoproteomic affinities, direct binding of GSK3011724A to purified KasA was also determined using surface plasmon resonance (SPR) and found to be in the 10–20 nM range (Supplementary Fig. 4). A much weaker apparent dissociation constant was observed for both Pks10 and Pks11 (*K*_d_^app^ 1.4 μM). In addition, we compared the results of the active compound GSK3011724A with a structurally related inactive analogue **2** (MIC>125 μM). The *K*_d_^app^ for this inactive molecule was determined to be 4.5 μM for KasA, which corresponds to a ∼490-fold window for KasA between active and inactive compound. There was no binding observed for Pks10 and Pks11 by the inactive analogue **2** (Fig. 3b; Supplementary Data 5).

To evaluate the potential of GSK3011724A for interaction with human proteins, which could represent off-targets relevant for drug safety, we performed a similar set of chemoproteomics experiments with a human protein extract, generated by combining detergent (NP40) lysates from HEK293 cells, K562 cells and placenta tissue (Supplementary Data 6). Only a single protein showed moderate competition by GSK3011724A, NQO2, with an apparent dissociation constant of 4–6 μM. NQO2 is an off-target of many small molecules drugs^24^. Other potential off-targets may exist that are not expressed in the cells and tissues used for the profiling, and the compound may exhibit binding modes, which are inconsistent with its immobilization. However, taken together, the chemoproteomics data demonstrate that GSK3011724A is a highly potent and selective inhibitor of KasA with no discernible off-targets in the above human cell lines and placenta tissue.

### Analysis of the KasA-GSK3011724A co-crystal structure

**Figure 4:**
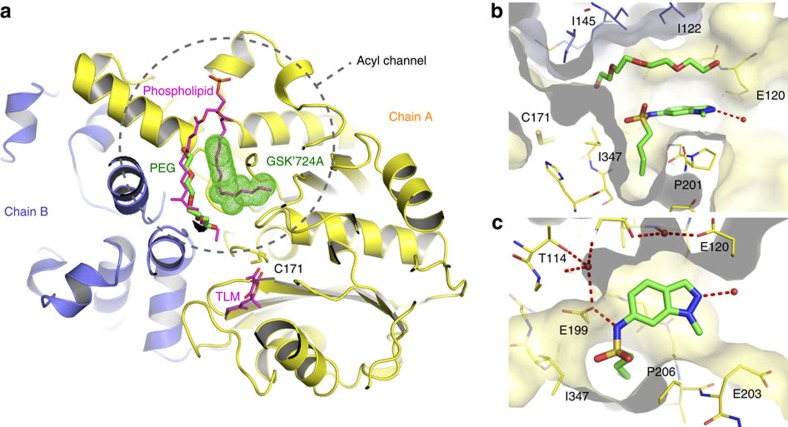
X-ray crystallographic analysis of KasA complexed with GSK3011724A. (**a**) Co-crystal structure of KasA dimer (yellow, blue) with GSK3011724A (green space filled) and PEG (green sticks) bound with the open conformation of the acyl channel. The active site cysteine C171 is shown in line format, as are an overlay of the TLM and a phospholipid ligand taken from PDB entry 4C72 (ref. 25). (**b**) GSK3011724A and PEG both shown in stick format filling the acyl channel. (**c**) Hydrogen bonds between GSK3011724A and E199 within the binding site.

Curious to understand the molecular details of GSK3011724A binding to KasA, a 2.13 Å co-crystal structure of the dimeric KasA-GSK3011724A complex was solved (Table 5). All literature inhibitors to date, such as TLM, reside in the malonyl substrate pocket close to the catalytic Cys171 residue^25^,^26^ (Fig. 4a). However, computational docking studies to place GSK3011724A into this site produced puzzling binding modes, which did not explain the roles of the key molecular features of GSK3011724A. The co-crystal structure reveals that, in contrast to TLM, GSK3011724A uniquely binds within the large acyl channel that normally accommodates the growing meromycolic acid chain, unexpectedly trapping the open conformation of the enzyme (Fig. 4a). GSK3011724A sits at the branch point of the acyl channel, a feature thought to confer specificity for ‘kinked' unsaturated substrates. Within this site, density for a long linear molecule, modelled as polyethylene glycol (PEG), is also consistently present and perfectly complements the space between the compound and the protein (Fig. 4b). This sandwich of PEG and GSK3011724A enables the large acyl cavity to be filled, occupying the volume of the branched phospholipid chains previously observed within this pocket^25^.

From the co-crystal structure, the structure–activity relationship of GSK3011724A is evident (Fig. 4b,c). The indazole ring lies across the flat hydrophobic surface formed by residues such as Gly200 and Pro201, and the amide of the sulfonamide makes a key hydrogen bond with Glu199. This anchors the ligand in an ideal position, allowing the kink (introduced by the sulfonamide linkage) to place the butyl chain into an orthogonal narrow lipophilic channel lined by residues such as Ile347, Ile202 and Phe239.

**Figure 5:**
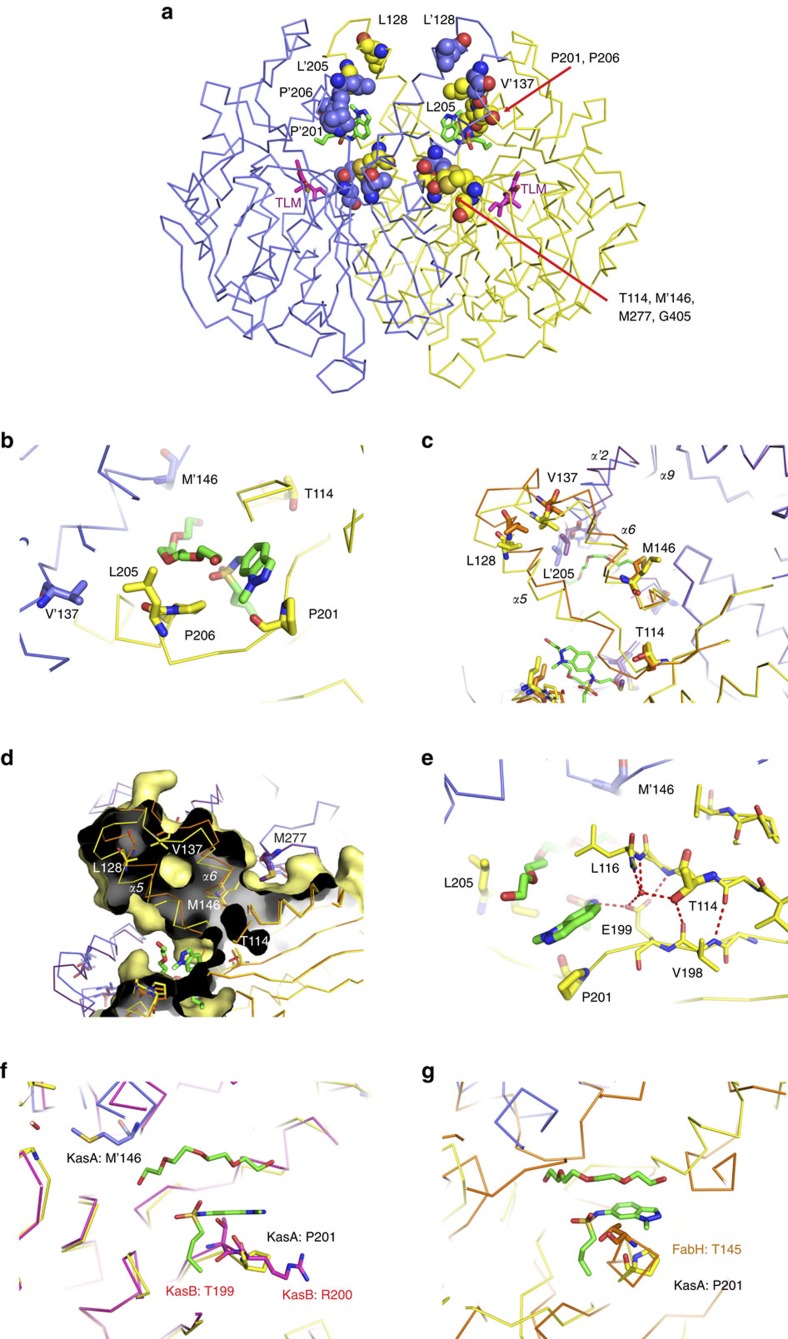
Impact of resistance-conferring mutations on the KasA-GSK3011724A crystal structure and structural comparison of KasA with KasB and FabH. (**a**) Mapping of residues where resistance mutations occur onto the dimeric KasA-GSK3011724A X-ray crystallographic complex. GSK3011724A (green stick) marks the acyl channel, whereas TLM (magenta) from PDB entry 4C72 (ref. 25) marks the malonyl pocket. (**b**) Residues P201 and P206 that confer resistance by direct interactions with GSK3011724A are shown, as are the proximity of other resistance positions visible. (**c**) A ribbon representation of the open and closed forms of KasA with resistance mutations highlighted. The open KasA-GSK3011724A dimer is shown in yellow and blue (chain A, B respectively). The apo, closed conformation (PDB entry 2WGD (ref. 26)) is shown in orange and purple. The HTH arm formed by α5–α6 and the α'2 from its dimer partner move substantially on transition between the open and closed states. Residues within these two regions of movement where resistance mutations are found are shown in stick format. GSK3011724A, PEG and other residues sensitive to resistance mutations are shown in line format. (**d**) Surface around Chain A within KasA-GSK3011724A complex shown to illustrate the role of M277 in the open and closed states. Colouring and representations identical to **c**. (**e**) Hydrogen bonding interactions around T114 within the open KasA-GSK3011724A complex. (**f**) Structural alignment of KasA-GSK3011724A (yellow and blue) with KasB (PDB entry 2GP6 (ref. 54)) (magenta). The indazole ring of GSK3011724A sits favourably over G200 and P201 in KasA. In KasB, the respective residues of T199 and R200 would not be able to accommodate this favourable binding mode. (**g**) Structural overlay of KasA-GSK3011724A (yellow and blue) with FabH (PDB entry 2QNZ (ref. 55)) (orange). In FabH, T145 would directly clash with the indazole ring of GSK3011724A, accounting for the compound selectivity for KasA.

The resistance-conferring mutations observed in Table 3 can be readily rationalized by the inhibitor binding site and MoA of GSK3011724A (Fig. 5a). Pro201 and Pro206 both lie within the acyl site and are in direct contact with the ligand (Fig. 5b). Exchange of proline for a hydrophilic serine residue (Pro201Ser) disfavours interactions with the aromatic indazole ring, whereas the Pro206Leu substitution introduces steric crowding of the ligand. The remaining mutations are found away from the inhibitor site, in regions likely to influence the conformational equilibrium and ease of transition between the closed and open state required for GSK3011724A inhibition. The α5 to α6 helix-turn-helix (HTH) arm of one KasA monomer, comprising of residues 115–145, must slide past its dimer counterpart (α′5–α′6) and the α′2 helix as the acyl cavity opens. Mutations Leu128Pro, Val137Ala, Met146Val and Leu205Pro are either within the HTH motif or the α2 helix and undergo substantial movement to the open form (Fig. 5c). Met277 also lies at the dimer interface, however, its position is fixed between the two states. This residue could be considered as the ‘ball' in a ‘ball and socket' joint around which the α5–α6 HTH pivots (Fig. 5d). Reducing the size of Met277 to valine may alter the movement and position of the HTH. Similarly, Thr114 sits at another fixed pivot point, the start of the first helix of the HTH (Fig. 5d). The positioning of Thr114 breaks the β-strand that proceeds it and anchors the HTH arm to its opposite β-strand partner Val198 via two hydrogen bonds (through a carbonyl-backbone interaction and by the interaction of the threonine side chain hydroxyl to the carbonyl of Val198) (Fig. 5e). The Thr114Ser resistance-conferring mutation would introduce more flexibility and may reduce the anchoring stability needed in this position.

In summary, these crystallographic insights complete a consistent picture of the molecular MoA of this inhibitor and provide a platform for rational optimization of the GSK3011724A scaffold as well as *de novo* structure-based drug design. Excitingly, this offers a fresh opportunity to target KasA at a site distinct from previous inhibitors and one that is able to achieve selectivity over other related β-ketoacyl synthases (FabH and KasB) involved in fatty acid biosynthesis, as key residues required for affinity are not conserved (Fig. 5f,g).

### *In vivo* target engagement of GSK3011724A and KasA

Although the evidence presented above provided a high level of confidence that KasA was the true biological target of GSK3011724A, there was still a need to confirm that target engagement was responsible for the potent *in vivo* activity. A selection of the *M. tuberculosis* spontaneous resistant mutants isolated against GSK3011724A were tested for their growth profile in mice using the acute model^11^ (Fig. 1d). While the two mutants showed an attenuated growth rate in untreated animals, their response to INH treatment remained essentially unchanged. Given the close relationship between KasA and InhA, the INH data were particularly significant, confirming that the observed resistance *in vivo* was not merely an artefact of the limited growth rate. In contrast with INH, the response with GSK3011724A against both strains with mutations mapping to *kasA* showed clear signs of resistance (Fig. 1d). Mutation Met277Thr appeared to impart complete resistance up to the maximum dose tested (140 mg kg^−1^), while mutation Pro201Ser gave a lesser response at 100 mg kg^−1^. These results support the MIC shifts observed *in vitro* and furthermore provide a critical link between the target identification and *in vivo* validation of KasA.

## Discussion

On-going efforts to combat drug-resistant TB have taken many forms including the re-purposing of broad spectrum antibacterials, target-based programs on mycobacterial enzymes, efforts to optimize or re-invent known TB drugs (like INH and RIF), and phenotypic screening approaches, which have all been widely reported^4^,^27^,^28^,^29^,^30^,^31^,^32^. In this work, through a variety of *in vitro* and *in vivo* experiments, a new chemical scaffold, exemplified by GSK3011724A, has been identified to specifically target an integral component of mycolic acid biosynthesis, KasA (ref. 18). Mycolic acids are unique and fundamental components of the mycobacterial cell wall and KasA is essential in *M. tuberculosis*^13^.

Mycolic acid biosynthesis involves two distinct fatty acid synthesis pathways. The FAS-I system is required for *de novo* fatty acid synthesis, where a single, multifunctional polypeptide generates short chain fatty acyl-CoA esters. FabH, a β-ketoacyl ACP synthase, forms a pivotal link between FAS-I and FAS-II, condensing C_14_-CoA (generated by FAS-I) and malonyl-AcpM producing C_16_-AcpM (ref. 15). This product is channelled to KasA of the FAS-II system^33^. The FAS-II system is comprised of four enzymes acting in a consecutive cycle: KasA and KasB, condensing enzymes^18^; MabA, a keto-reductase^34^; HadABC, a dehydratase^35^; and InhA, an enoyl-reductase^36^. The FAS-II system enables fatty acid elongation leading to meromycolic acids (C_56_), which are then condensed with C_26_-CoA (from FAS-I) by the polyketide synthase Pks13 (refs 37, 38), followed by reduction, culminating in the production of mature mycolic acids^39^. The lipidomics experiments showing accumulation of FAMEs and depletion of MAMEs confirms that GSK3011724A specifically inhibits FAS-II and mycolic acid biosynthesis (Fig. 2a,b; Table 4; Supplementary Fig. 2).

Inhibitors of KasA have been reported in the literature^14^,^40^,^41^. Most notable among these is TLM, which is known to inhibit all three mycobacterial Kas enzymes: KasA, KasB and FabH^14^,^15^. Interestingly, GSK3011724A, unlike TLM and other Kas inhibitors, whether against *M. tuberculosis* or other bacteria (via FabH, FabB and FabF), displayed unique specificity, targeting only KasA (Fig. 2c). An explanation of this finding comes from close inspection of the GSK3011724A-KasA complex crystal structure, where specific changes in the acyl pockets of the related enzymes disfavour the unique binding mode of GSK3011724A (Figs 4 and 5). For example, the Gly200Arg and Pro201Thr changes from KasA to KasB no longer allow the indazole ring to sit favourably in the acyl pocket of KasB (Fig. 5f). This offers an explanation as to why the WGS data did not result in the identification of SNPs in KasB and why the resistance mutations in KasA often map to points of variation amongst the Kas enzymes.

Importantly, GSK3011724A, despite representing an un-optimised screening hit, gives at least one order of magnitude greater potency relative to the most studied KasA inhibitor, TLM, and its favourable PK properties allowed for the critical *in vivo* experiments described in this work. The data obtained from murine assays (both acute and chronic infection) exemplify the credible potential of KasA inhibitors to give significant efficacy and, to our knowledge, offer the first *in vivo* validation of KasA as a drug target (Fig. 1d). Together with the promising drug-like profile of GSK3011724A, these data provide significant confidence for the future exploration of KasA as a drug target, inhibitors of which could become key players in the development of new anti-tubercular drugs and will be explored in a future publication.

## Methods

Synthetic and characterization details for the compounds and the SPR experiment described herein can be found in Supplementary Methods. All animal studies were ethically reviewed and carried out in accordance with European Directive 2010/63/EU and the GSK Policy on the Care, Welfare and Treatment of Animals. The human biological samples were sourced ethically and their research use was in accordance with the terms of informed consent.

### Assessment of acute and chronic efficacy in murine TB models

INH was purchased from Sigma-Aldrich and prepared freshly in distilled water. GSK3011724A was prepared freshly in 1% aqueous methylcellulose. The assessment of the chronic and acute efficacy in murine TB models was performed using specific pathogen-free, 8–10 week-old female C57BL/6 mice purchased from Harlan Laboratories and allowed to acclimate for 1 week and kept under controlled conditions in a P3 high-security facility with unlimited sterile food and water.

In the acute model^11^, mice were intratracheally infected with *M. tuberculosis* H37Rv wild-type (H37Rv WT) 100,000 c.f.u. for each mouse, and lungs harvested on day 9. GSK3011724A and INH were administered daily for 8 consecutive days, starting on day 1 after infection. In the chronic model^42^, mice (*n*=2 mice at each dose level) were intratracheally infected with 100 c.f.u. for each mouse; INH or GSK3011724A was administered daily for 8 consecutive weeks, starting 6 weeks after infection. Lungs were harvested 24 h after the last administration in both assays. All lung lobes were aseptically removed, homogenized and frozen. Homogenates were unfrozen and plated in 10% OADC-7H11 medium supplemented with activated charcoal (0.4%) and grown for 18–25 days at 37 °C. Non-linear fitting was performed with the dose–response data (log c.f.u. versus dose) and the dose in mg kg^−1^ that reduced lung bacterial burden by 99% with respect to untreated mice was estimated (ED_99_). Mice were supervised every day under a protocol paying attention to weight loss, apparent good health (bristled hair and wounded skin) and behaviour (signs of aggressiveness or isolation). Animals were euthanized by CO_2_ inhalation.

### MIC determination and resistant mutant generation

*M. bovis* BCG strain Pasteur and derivatives were cultured at 37 °C and 5% CO_2_ in static liquid or solid medium. Liquid medium contained Middlebrook 7H9 (Difco) supplemented with 0.05% (v/v) Tween-80, 10% (v/v) Middlebrook ADC and 0.25% (v/v) glycerol. Solid medium contained Middlebrook 7H11 agar (Difco) with 10% (v/v) Middlebrook OADC and 0.5% (v/v) glycerol. Where applicable, 25 μg ml^−1^ Kanamycin was added to the liquid or solid media to select for mycobacterial plasmids. The constructs pMV261, pMV261-Mt-*kasA*, pMV261-Mt-*kasB*, pMV261-Mt-*fabH*, pMV261-Mt-*inhA*, pMV261-Mt-*hadABC* and pMV261-Mt-*mabA* were electroporated into *M. bovis* BCG^14^,^15^,^16^,^17^. Wild-type *M. bovis* BCG electrocompetent cells were prepared by pelleting a mid-log culture and washing with decreasing volumes of ice-cold 10% (v/v) glycerol. The cells were incubated on ice with 1 μg plasmid DNA before being transferred to a 0.1 cm electrode-gap electroporation cuvette and subjected to a single pulse of 1.8 kV. Cells were recovered in liquid media overnight at 37 °C and selected on solid medium containing the appropriate antibiotic.

The MIC of GSK3011724A was determined by plating 10^4^, 10^3^, 10^2^ and 10^1^ cells from a mid-log culture of *M. bovis* BCG on solid medium containing increasing concentrations of compound in a dose–response format. The MIC was defined as the concentration of compound that caused complete inhibition of bacterial growth. *M. bovis* BCG and *M. tuberculosis* spontaneous resistant mutants were generated by plating 10^8^ cells from a mid-log phase culture on solid media containing either 5 × , 10 × or 20 × MIC of GSK3011724A. Potentially resistant colonies were inoculated into liquid media, cultured to mid-log growth phase, and selected on solid media containing 5 × MIC of GSK3011724A to confirm phenotypic resistance. The MIC of the resistant *M. tuberculosis* mutant strains against GSK3011724A was determined either using the MABA resazurin assay^43^ or by serial dilution and agar plating.

### Sequencing of resistant mutants

Wild-type *M. bovis* BCG and the *M. bovis* BCG GSK3011724A-resistant mutants were characterized by WGS^6^,^9^. Briefly, purified genomic DNA was prepared for sequencing using the Nextera DNA Sample Preparation Kit (Illumina). The DNA libraries were purified and quantified using Agencourt AMPure XP beads (Beckman Coulter Genomics) and Quant-iT PicoGreen dsDNA kit (Life Technologies), respectively. Fragment sizes were determined using an Agilent Technologies 2100 Bioanalyzer with a High Sensitivity DNA chip. Following the MiSeq preparation guide, the libraries were sequenced on a MiSeq Benchtop Sequencer using the MiSeq Reagent Kit v2, 300 cycles. Reads were aligned to the reference genome *M. bovis* BCG Pasteur 1173P2 (accession: NC_008769.1).

The *kasA* gene (Rv2245) from *M. tuberculosis* GSK3011724A-resistant mutants was sequenced specifically. The gene was amplified by PCR using 0.5 μM of the flanking primers 5′- aggacaagtacggcgtcaag (forward) and 5′- gtaaccagctccgtcattg (reverse). The amplification was performed with 0.02 U μl^−1^ of KOD Xtreme Hot Start DNA Polymerase (Merck Millipore) and 0.4 mM each of dNTP. PCR conditions were: 2 min at 94 °C, followed by 30 cycles of 98 °C for 10 s, 66 °C for 60 s and 68 °C for 10 s. The PCR product was purified from the band of a 0.8% agarose gel (Seakem GTG Agarose, Lonza) with Illustra GFX PCR DNA and Gel Band Purification Kit, GE Healthcare. The purified product was used for *kasA* automated sequencing (using the primers: Forward 1, cgaagattgagtcggagaac ; Reverse 1, cttccatatcggtccgactc ; Forward 2, gtcaagatcggcggtcac ; Reverse 2, cagacttcggcgcgtaca ; Forward 3, gtgatcggtctgcagcttg ; Reverse 3, ctcctccgtctcgatgag ; Forward 4, ctcatcgagacggaggag ; Reverse 4, cgttgggcatgatcatctg ; Forward 5, tgtacgcgccgaagtctg ; Reverse 5, gatcccacttggtgacgaac ). DNA sequencing reactions were performed with a BigDye terminator V3.1 cycle sequencing kit (Applied Biosystems).

### Synthesis of FAMEs and MAMEs

The whole-cell effect of GSK3011724A was studied by treating *M. bovis* BCG cultures (10 ml) at an OD_600nm_ of 0.4–0.6 with a dose-dependent increase in drug for 20 h before labelling using 1 μCi ml^−1^ [1-^14^C]sodium acetate (37 MBq, PerkinElmer) for a further 24 h at 37 °C. The total FAMEs and MAMEs were extracted^14^,^44^. Briefly, cells were pelleted and incubated overnight in 2 ml of 5% tetrabutylammonium hydroxide at 100 °C. The following day, 4 ml of dichloromethane was added with 300 μl of iodomethane and 2 ml of water and mixed for 30 min. The reaction was centrifuged and the upper aqueous layer discarded. Water (3 ml) was added to the lower organic layer, mixed and centrifuged as before and repeated once more. The organic layer was evaporated to dryness and the methyl esters re-dissolved in diethyl-ether (4 ml) and transferred to a fresh tube. The diethyl-ether was evaporated and 200 μl of dichloromethane used to re-dissolve the extracted methyl esters. The total FAMEs and MAMEs were analysed by TLC, using equal counts (c.p.m.) and exposed to Kodak X-Omat film. Quantification of labelled FAMEs and MAMEs was determined by excising spots directly from the TLC plates and subjecting them to scintillation counting using 10 ml of EcoScintA.

### Extraction of cell wall-bound MAMEs and lipids

*M. bovis* BCG was drug-treated and labelled as described above. The cell wall-bound MAMEs, apolar and polar phospholipids were extracted and analysed^44^. Briefly, the cell pellet was extracted four times using 4 ml of chloroform:methanol:water (10:10:3, v/v/v), retaining the delipidated cell pellet (for analysis of cell wall-bound mycolic acids) and collecting the solvent extract sequentially, which was combined and dried. To the dried extract, 4 ml of chloroform:methanol:water (10:10:3, v/v/v) was added, followed by chloroform (1.75 ml) and water (0.75 ml), and the entire mixture centrifuged and the lower organic layer recovered. The lower organic layer was washed twice using chloroform:methanol:water (2 ml, 3:47:48, v/v/v) and dried to provide a total lipid extract, which was re-dissolved in chloroform:methanol (2:1, v/v) and an aliquot subjected to scintillation counting using 10 ml of EcoScintA. The total lipid extract was further partitioned between the phases arising from methanol:0.3% NaCl (2 ml, 100:10, v/v) and 2 ml of petroleum-ether (60–80 °C). The entire contents were mixed on a blood rotor, centrifuged and the upper layer collected. The lower layer was re-extracted using 2 ml of petroleum-ether (60–80 °C). The combined petroleum-ether layers were evaporated to afford the crude apolar lipids. To the lower organic layer, 2.3 ml of chloroform:methanol:0.3% NaCl (50:100:40, v/v/v) was added, followed by 750 μl of chloroform:methanol:0.3% NaCl (50:100:40) and a further 1.3 ml of chloroform and 1.3 ml 0.3% NaCl. The entire contents were mixed, centrifuged and the lower layer recovered and dried to afford the crude polar lipids. The apolar and polar lipids were re-dissolved in chloroform:methanol (2:1, v/v) and an aliquot subjected to scintillation counting using 10 ml of EcoScintA. The total lipid extracts and apolar/polar lipids were analysed by TLC in the following solvent systems using equal counts (as stated) before being exposed to Kodak X-Omat film. Apolar lipids were resolved using three solvent systems A: first direction, petroleum-ether (60:80 °C)):ethyl acetate (98:2, v/v, thrice); second direction, petroleum-ether (60:80 °C):acetone (98:2, v/v). Solvent system B: first direction, petroleum-ether (60:80 °C):acetone (92:8, v/v, thrice); second direction, toluene:acetone (95:5, v/v). Solvent system C: first direction, chloroform:methanol (96:4, v/v); second direction, toluene:acetone (80:20, v/v). The polar lipids were analysed using chloroform:methanol:ammonium hydroxide:water (65:25:0.5:3.6, v/v/v). The total lipid extract was analysed using chloroform:methanol:concentrated ammonium hydroxide (80:20:2, v/v/v) to reveal TDM and TMM. The recovered delipidated cells were used to analyse cell wall-bound mycolic acids following release using 5% tetrabutylammonium hydroxide at 100 °C and methylation as described above for total FAMEs and MAMEs. The recovered cell wall-bound MAMEs were analysed by TLC, using an equal aliquot (5%) and exposed to Kodak X-Omat film.

Original scans of all TLCs are shown in Supplementary Fig. 5.

### KasA activity assay

Recombinant FabD, holo-AcpM and C_16_-AcpM were overexpressed in C41 (DE3) *Escherichia coli* cells from pET28a-*fabD* and pET28a-*acpM* and purified^45^. Briefly, cells were resuspended in buffer (50 mM potassium phosphate, pH 7.5, 0.5 M NaCl and 10 mM (AcpM)/25 mM (FabD) imidazole) containing DNAse, Complete protease inhibitor-cocktail tablets (Roche) and 0.1 mg ml^−1^ lysozyme. Cells were disrupted by 6 passes through a French Press and the clarified lysate was loaded onto a pre-equilibrated (with buffer) Ni^2+^-charged 1 ml His-Trap column. A step gradient of imidazole (50–1,000 mM) was used to wash and elute the recombinant protein. Recombinant AcpM was dialysed into 0.1 M Tris pH 7.5, 500 mM NaCl and loaded onto a column containing 1 ml Thiopropyl-Sepharose 6B. Acyl-AcpM was collected in the flow through. Holo-AcpM was eluted with an increasing concentration of β-mercaptoethanol (5–100 mM). All purified enzymes were dialysed firstly against 50 mM Tris pH 7.5, 50 mM NaCl, 10% (v/v) glycerol, 2 mM EDTA and secondly against the same buffer without EDTA.

The *E. coli* expression plasmid, pET28a-*kasA* (ref. 18), was transformed into *E. coli* BL21 (DE3). A resulting single colony was used to inoculate an overnight culture, which was subsequently used to inoculate 1 l LB broth, 1% (w/v) glucose, 50 μg ml^−1^ Kanamycin. The culture was incubated at 37 °C, 180 rpm, until OD_600nm_ reached 0.4–0.6. The culture was cooled to 16 °C and induced with 1 mM IPTG. Growth was continued for 20 h at 16 °C, 180 r.p.m., and then the cells were harvested by centrifugation. The cell pellet was resuspended in buffer (50 mM potassium phosphate, pH 7.45, 0.5 M NaCl and 10 mM imidazole) containing DNAse, RNAse, Complete protease inhibitor-cocktail tablets (Roche) and 0.1 mg.ml^−1^ lysozyme. Cells were disrupted by 6 passes through a French Press at 1 ksi and centrifuged at 15,000 r.p.m., 40 min at 4 °C. The supernatant was loaded onto a pre-equilibrated (with buffer) Ni^2+^-charged 1 ml His-Trap column. The column was washed extensively using buffer and the proteins were eluted with a step-wise gradient of imidazole (50, 100, 150, 200, 350 and 500 mM). SDS–PAGE was used to detect the presence of purified KasA, which was dialysed against 50 mM Tris.HCl, pH 7.5, 300 mM NaCl, 10% (v/v) glycerol and stored at −20 °C.

The assay for mycobacterial KasA activity was performed^18^. Assay components were mixed together in a batch fashion, before equally dividing according to the number of assays performed. The amounts stated correspond to a single reaction. Holo-AcpM (40 μg), in 200 mM potassium phosphate pH 7.0, 5 mM β-mercaptoethanol and a final volume of 40 μl, was incubated on ice for 30 min. [2-^14^C]Malonyl-CoA (0.05 μCi, 1.85 kBq, PerkinElmer) and 50 ng FabD were added and the reaction was incubated at 37 °C for 1 h. 42.5 μg C_16_-Acpm:Holo-AcpM mix (42.5 μg) was added with 200 mM potassium phosphate and 5 mM β-mercaptoethanol to a final volume of 89 μl. The reaction mix was aliquoted into 1.5 ml microcentrifuge tubes, according to single assay conditions. An aliquot of KasA (0.25 μg) (or replaced with an equal volume of buffer) was added and the reactions were incubated at 37 °C for 1.5 h. The reaction was quenched with 2 ml of freshly prepared reducing solution: 5 mg ml^−1^ NaBH_4_ in 0.1 M K_2_HPO_4_, 0.4 M KCl, 30% (v/v) tetrahydrofuran. The reaction was incubated overnight at 37 °C. The reduced β-ketoacyl product was extracted twice using 2 ml of water-saturated toluene, and the combined organic phase washed thrice using 2 ml of toluene-saturated water. The organic layer was transferred to a scintillation vial and dried. The radiolabelled product was quantified by liquid scintillation counting using 10 ml of EcoScintA.

### Chemoproteomics

The chemoproteomic inhibition binding experiments were performed as previously described^19^,^20^. Briefly, sepharose beads were derivatized with **1**, the GSK3011724A-tagged analogue, at 2 mM compound concentration. Beads were washed and equilibrated in lysis buffer (50 mM Tris-HCl, pH 7.4, 0.4% Igepal-CA630, 1.5 mM MgCl_2_, 5% glycerol, 150 mM NaCl, 25 mM NaF, 1 mM Na_3_VO_4_, 1 mM dithiothreitol (DTT) and one Complete EDTA-free protease inhibitor tablet (Roche)). The equilibrated beads were incubated at 4 °C for 1 h either with 0.1 ml (0.3 mg) *M. bovis* BCG extract or with 1 ml (5 mg) mixed HEK293/K562/Placenta extract, which was pre-incubated with compound or DMSO (vehicle control). Beads were transferred either to Filter plates (Durapore (PVDF membrane, Merck Millipore)) or to disposable columns (MoBiTec), washed extensively with lysis buffer and eluted with SDS sample buffer. Proteins were alkylated, separated on 4–12% Bis-Tris NuPAGE (Life technologies) and stained with colloidal Coomassie. Gel lanes were cut into three slices and subjected to in-gel digest using LysC for 2 h and trypsin overnight^19^. Digestion, labelling with TMT isobaric mass tags, peptide fractionation and mass spectrometric analyses were performed^19^,^46^. Proteins were quantified by isobaric mass tagging and liquid chromatography-tandem mass spectrometry (LC-MS/MS). The proteins.fasta file for *M. bovis* BCG was downloaded (11th May 2011) from http://genome.tbdb.org/annotation/genome/tbdb/MultiDownloads.html and supplemented with the sequences of bovine serum albumin, porcine trypsin and mouse, rat, sheep and dog keratins. Decoy versions of all proteins were created and added. The search database contained a total of 11,492 protein sequences, 50% forward, 50% reverse. Protein identification and quantification was performed^47^. Proteins identified with >1 unique peptide matches were considered for further data analysis. Apparent dissociation constants were determined by taking into account the protein depletion by the beads^19^. Raw data tables for the chemoproteomics experiments can be found in the Supplementary Data 1–6.

The *M. bovis* BCG extracts were prepared as follows: *M. bovis* BCG was cultured in 7H9 medium without glycerol and supplemented with 2% (w/v) glucose and 0.025% (v/v) tyloxapol at 37 °C for 8–10 days to reach an OD_600nm_ of 0.8–1.0. The culture was centrifuged and the pellet was washed with PBS and 0.025% (v/v) tyloxapol. The pellet was resuspended in lysis buffer (0.4% (v/v) Igepal was replaced with 0.8 (v/v)% NP40) and sonicated for three cycles at 50% amplitude for 30 s (Sonics-VibracellTM) in ice. This lysate was ultracentrifuged at 4 °C for 60 min and the cellular debris was discarded.

### KasA protein production and structure determination

The overexpression plasmid, pET28a-*kasA*, was transformed into *E. coli* BL21 (DE3) cells. A single colony was used to inoculate 100 ml LB broth containing 50 μg ml^−1^ Kanamycin and 1% (v/v) glucose. Cells were cultured overnight at 30 °C, 240 r.p.m. A 20 l Biolaffite fermenter containing 15 l of Overnight Express Instant TB (Merck), 1% (v/v) glycerol, 50 μg ml^−1^ Kanamycin and 20 ml of antifoam (DC1520) was inoculated with the overnight culture to 2% (v/v). The culture was grown at 37 °C, 340 r.p.m., 12 l min^−1^ air flow. The fermenter was cooled to 25 °C when the OD_600nm_ reached 2.4. The culture was incubated for 20 h before harvesting the cells. The cell pellet was resuspended in 5 ml g^−1^ cells in Buffer A (50 mM Tris, 500 mM NaCl, 10% (v/v) glycerol, 2 mM DTT, pH 8.5, with 1 mg ml^−1^ lysozyme, Protease inhibitor-cocktail set III (Sigma) and 10 μl Benzonase). The sample was lysed by sonication on ice for 10 min (10 s on, 10 s off). The lysate was centrifuged at 18,000 r.p.m., 4 °C for 30 min. The supernatant was loaded onto a pre-equilibrated Ni^2+^-charged 10 ml His-Trap column. The column was washed back to baseline with Buffer A and the protein was eluted using a linear gradient over 20 column volumes using Buffer B (Buffer A containing 500 mM imidazole). KasA was further purified by gel filtration in Buffer A using a Superdex 200 column.

KasA was co-crystallized with GSK3011724A using protein at 10.6 mg ml^−1^ and ligand at a nominal concentration of 30 mM in 100+100 nl sitting drops at 20 °C. The well solution was 8% (w/w) isopropanol, 0.2 M NaCl, 10 mM tris(2-carboxyethyl)phosphine (TCEP). Crystals were cryoprotected using 30% (v/v) glycerol before flash freezing in liquid nitrogen. Data from a single crystal was collected at the Diamond Synchrotron Radiation Facility (i04) and processed in P3_1_ to 2.13 Å using XDS (within AUTOPROC [Global Phasing Limited])^48^ and AIMLESS^49^. A molecular replacement solution was determined with a previously collected in house structure using Phaser^50^. The P3_1_ cell (*α*=*β*=90°, *γ*=120°, *a*=*b*=77.338 Å, *c*=147.675 Å) has two molecules in the asymmetric unit that form a dimer. Model building and refinement of the KasA structures was carried out using alternating rounds of COOT^51^ for manual model building and REFMAC^52^ for maximum likelihood refinement via CCP4 (ref. 53). As the data was merohedrally twinned, TWIN refinement within REFMAC was used, with the refined twin fraction being 39%. A clear difference in density for GSK3011724A, and also for a PEG-like molecule was present in both chains in the dimer. Whilst PEG was not explicitly added to the wells, this was present in adjacent crystallization conditions and we believe there may be trace PEG present. Alternatively, the linear molecule may be residual lipid present in the protein, although *apo*-structures do not contain this lipid and the protein is crystallized in the closed conformation even in PEG conditions. A stereo diagram is displayed in Supplementary Fig. 6. Statistics for the data collection and refined co-ordinates are given in Table 5.

### Data availability

The atomic co-ordinates and structure factors reported in this paper have been deposited in the Protein Data Bank with the code 5LD8. Data is available upon request from the corresponding authors.

## Additional information

**How to cite this article:** Abrahams, K. A. *et al*. Identification of KasA as the cellular target of an anti-tubercular scaffold. *Nat. Commun.* 7:12581 doi: 10.1038/ncomms12581 (2016).

## Supplementary Material

Supplementary Information Supplementary Figures 1-6, Supplementary Table 1 and Supplementary Methods

Supplementary Data 1 Competition binding of *M. bovis* BCG extracts to GSK3011724A-derivatised beads MS/MS data for depletion of proteins by the GSK3011724A-tagged analogue (1) probe matrix in *M. bovis* BCG cell extracts, and resulting correction factors for calculation of apparent dissociation constants from IC50 values (Sample volume, 0.1 mL; protein extract, 0.25 mg/sample)

Supplementary Data 2 Identification of proteins from *M. bovis* BCG extracts binding to GSK3011724A-derivatised beads MS/MS data for depletion of proteins by the GSK3011724A-tagged analogue (1) probe matrix in *M. bovis* BCG cell extracts, and resulting correction factors for calculation of apparent dissociation constants from IC50 values (Sample volume, 0.1 mL; protein extract, 0.3 mg/sample; 2 mM compound concentration on beads)

Supplementary Data 3 Generation of KasA IC50 values for GSK3011724A Proteins identified with GSK3011724A-tagged analogue (1)-matrix in *M. bovis* BCG cell extracts. Generation of IC50 values for test compound GSK3011724A (Sample volume, 0.1 mL; protein extract, 0.3 mg/sample; 2 mM compound concentration on beads)

Supplementary Data 4 Generation of Pks10 and Pks11 IC50 values for GSK3011724A Proteins identified with GSK3011724A-tagged analogue (1)-matrix in *M. bovis* BCG cell extracts. Generation of IC50 values for test compound GSK3011724A (Sample volume, 0.1 mL; protein extract, 0.3 mg/sample; 2 mM compound concentration on beads)

Supplementary Data 5 Competition binding of *M. bovis* BCG extracts to GSK3011724A-derivatised beads in the presence of an inactive GSK3011724A analogue Proteins identified with GSK3011724A-tagged analogue (1)-matrix in *M. bovis* BCG cell extracts. Generation of IC50 values for test compound 2, the inactive analogue (Sample volume, 0.1 mL; protein extract, 0.3 mg/sample; 2 mM compound concentration on beads)

Supplementary Data 6 Competition binding of human cell lines with GSK3011724A-derivatised beads. Proteins identified with GSK3011724A-tagged analogue (1)-matrix in lysates from HEK293 cells, K562 cells, and placenta tissue. Generation of IC50 values for test compound GSK3011724A (Sample volume, 1.0 mL; protein extract, 5.0 mg/sample; 2 mM compound concentration on beads).

## Figures and Tables

**Table 1:** *In vitro* profile of GSK3011724A.

**Assay**	**Value**
MIC H37Rv	0.8 μM
HepG2 IC_50_	>100 μM
ChromLogD_7.4_	3.8
Kinetic Solubility	>550 μM
hERG IC_50_	>50 μM
CYP3A4 IC_50_	>40 μM
CYP1A2 IC_50_	>50 μM
CYP2C9 IC_50_	12.6 μM
CYP2D6 IC_50_	>50 μM
18 Gram-positive and Gram-negative species	>128 μg ml^−1^
Plasma protein binding (mouse)	76.9%
Plasma protein binding (human)	91.8%
Cli (mouse)	6.1 ml min^−1^ g^−1^
Cli (human)	0.95 ml min^−1^ g^−1^

**Table 2:** *In vivo* pharmacokinetic profile of GSK3011724A.

**Target dose (mg kg**^**−1**^**)**	***C***_**max**_ **(ng ml**^**−1**^**)**	***C***_**max**_**/Dose ng ml**^**−1**^ **(mg kg**^**−1**^**)**	***t***_**max**_ **(h)**	**AUC**_**0-*****t***_ **(ng h ml**^**−1**^**)**	**DNAUC ng h ml**^**−1**^ **(mg kg**^**−1**^**)**
10*	917±279	84.7±21.5	0.25–0.75	1099±152	104±19.1
25	1392±424	63.9±19.4	0.5	2279±581	107±27.3
50	12500±153	250±23.1	0.25–1.0	32549±3009	667±64.2
100	10570±572	120±6.49	0.75–1.0	57179±7559	667±57.2
200	33000±7017	165±35.1	2.0	251437±37054	1272±188

AUC, area under the curve; *C*_max_, maximum concentration measured; DNAUC, dose-normalized area under the curve.

Pharmacokinetic parameters estimated for GSK3011724A after a single oral gavage administration to female C57BL/6J mice (*n*=3) at different doses. Mice dosed above 400 mg kg^−1^ were withdrawn after the second administration of GSK3011724A due to poor clinical status. Standard deviation (s.d.) of the mean is also included.

^*^Mean and s.d. values calculated from *n*=3 mice.

**Table 3:** Sequencing of spontaneous resistant mutants identifies SNPs in *kasA*.

**Organism**	**Mutant**	**Frequency of SNP**	**Genome position of SNP**	**Base change**	**Amino-acid substitution**
*M. bovis* BCG	1	75%	2497100	aCc/aGc	T114S
		2.44%	2497376	cCc/cTc	P206L
	2	100%	2497142	cTg/cCg	L128P
	3	100%	2497373	cTg/cCg	L205P
	4	100%	2497376	cCc/cTc	P206L
	5	100%	2497195	Atg/Gtg	M146V
	6	100%	2497589	aTg/aCg	M277T
*M. tuberculosis*	1	—	2518524	gTg/gCg	V137A
	2	—	2518524	gTg/gYg	V137A/V
	3	—	2518524	gTg/gCg	V137A
	4	—	2518524	gTg/gCg	V137A
	5	—	2518715	Ccc/Tcc	P201S
	6	—	2518715	Ccc/Tcc	P201S
	7	—	2518715	Ccc/Tcc	P201S
	8	—	2518715	Ccc/Tcc	P201S
	9	—	2518943	Atg/Gtg	M277V
	10	—	2518944	aTg/aCg	M277T
	11	—	2519327	Ggc/Agc	G405S

SNP, single-nucleotide polymorphism.

WGS and sequencing of *kasA* from GSK3011724A-resistant mutants of *M. bovis* BCG and *M. tuberculosis*, respectively, revealed *kasA*-specific mutations. For the WGS of *M. bovis* BCG mutants, the frequency of SNP is shown. This corresponds to the percentage of SNP in the cell population from which the genomic DNA was extracted. The *kasA* SNP frequency in the *M. tuberculosis* mutants cannot be deduced due to the method of analysis used. The genomic location of the mutations is stated. The missense mutations are represented in codons, where the capital letter identifies the base change. The ‘Y' indicates a mixed population of ‘C' or ‘T' bases. The resulting amino-acid substitutions are listed.

**Table 4:** Quantification of inhibition of mycolate containing products by GSK3011724A.

**Inhibitor**	**Inhibitor concentration (μM)**	**FAMEs**		**α-MAMEs**		**k-MAMEs**		**TDM**		**TMM**	
		**c.p.m.**	**% Relative to control**	**c.p.m.**	**% Relative to control**	**c.p.m.**	**% Relative to control**	**c.p.m.**	**% Relative to control**	**c.p.m.**	**% Relative to control**
Control	0	9,796	100	4,029	100	7,641	100	3,149	100	1,425	100
GSK3011724A	0.25	16,207	165	3,438	85	7,147	94	ND	ND	ND	ND
	0.5	17,780	182	3,026	75	6,651	87	864	27	911	64
	1	16,478	168	2,488	62	4,925	64	874	28	893	63
	2	18,268	186	1,806	45	4,407	58	ND	ND	ND	ND
	4	ND	ND	ND	ND	ND	ND	652	21	951	67
Control	0	10,231	100	4,156	100	6,722	100	—*	—*	—*	—*
TLM	95	15,334	150	2,721	65	3,727	55	817	26	704	49
INH	1.5	16,926	165	1,426	34	1,808	27	642	20	552	39

α-MAMEs, α-mycolate acid methyl esters; FAMEs, fatty acid methyl esters; INH, isoniazid; k-MAMEs, keto-mycolic acid methyl esters; ND, not determined; TDM, trehalose dimycolate; TLM, thiolactomycin; TMM, trehalose monomycolate.

Quantification of [^14^C]-labelled products were determined by excising spots directly from TLC plates (Fig. 2a, left and middle panels, and Fig. 2b) and subjecting them to scintillation counting using 10 ml of EcoScintA.

^*^Control values as reported above.

**Table 5:** Data collection and refinement statistics.

**X-ray diffraction data**	**KasA/GSK3011724A**
*Data collection*	
Space group	P3_1_
Cell dimensions	
*a*, *b*, *c* (Å)	77.34, 77.34, 147.68
*α*, *β*, *γ* (°)	90.000, 90.000, 120.000
Resolution (Å)	66.98–2.13(2.38–2.13)
*R*_merge_	0.053 (0.474)
*ccI1/2*	0.998 (0.822)
*I*/σ*I*	16.9 (3.0)
Completeness (%)	98.6 (97.3)
Redundancy	4.3(4.0)
Wilson B-factor	34.16
*Refinement*	
Resolution (Å)	66.98–2.13
No. reflections	235866 (60834)
No. uniq reflections	54699 (15385)
*R*_work/_ *R*_free_	0.162/0.186
No. atoms	6470
Protein	6041
Ligand/ion	36/28
Water	365
B-factors	
Protein	39.70
Ligand/ion	39.90/48.61
Water	43.92
R.m.s.d.	
Bond lengths (Å)	0.0044
Bond angles (°)	0.8045
Twin fraction	0.39

Values in parentheses are for the highest-resolution shell. Collection based on a single crystal.
