# Supplementary material for: Identification of KasA as the cellular target of an anti-tubercular scaffold
Source: Nat Commun. 2016 Sep 1;7:12581. doi: 10.1038/ncomms12581 (PMC5025758; doi:10.1038/ncomms12581)
Supplement: Supplementary Information — Supplementary Figures 1-6, Supplementary Table 1 and Supplementary Methods [file ncomms12581-s1.pdf]

Supplementary Information

Supplementary Figures

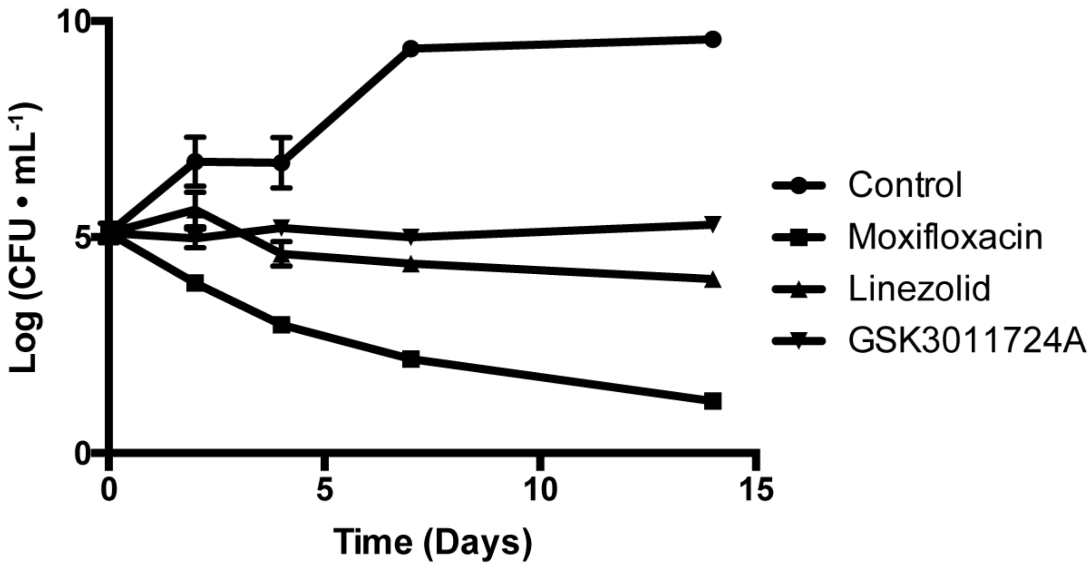

Supplementary Figure 1: *In vitro* death curve analysis of GSK3011724A. *M. tuberculosis* was treated with GSK3011724A at 0.5 µM (10 x MIC in liquid media), along with the bacteriostatic and bactericidal compounds, linezolid (17.8 µM) and moxifloxacin (1.5 µM), respectively (10 x MIC), and compared to untreated cells (control). CFU counts were performed in triplicate and analysed and displayed as mean ± standard deviation. GSK3011724A exhibited bacteriostatic activity in this *in vitro* system, despite its bactericidal activity *in vivo*.

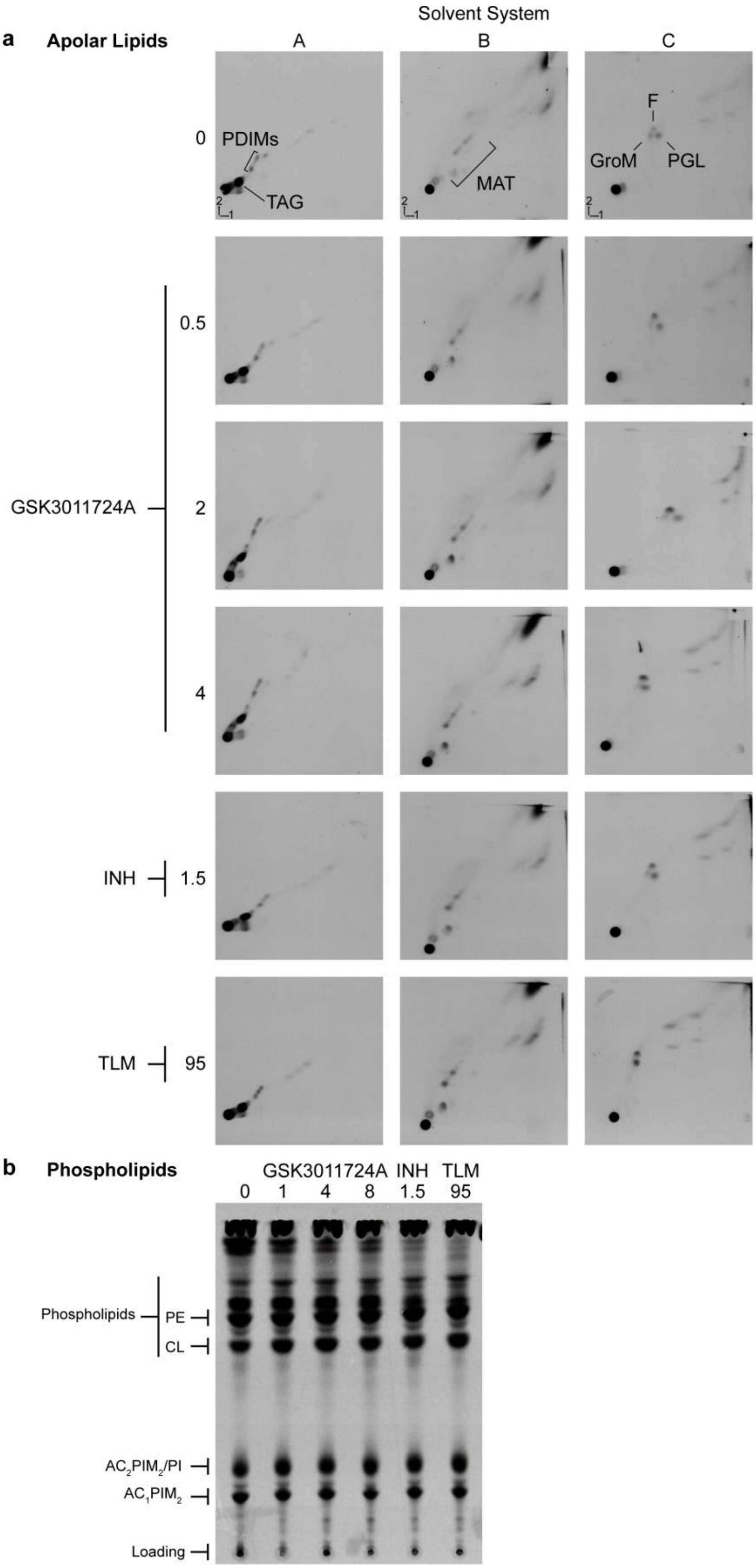

a
Apolar Lipids
Solvent System
A
B
C
0
PDIMs
TAG
MAT
F
GroM
PGL
0.5
GSK3011724A
2
4
INH
1.5
TLM
95
b
Phospholipids
GSK3011724A
INH
TLM
0
1
4
8
1.5
95
Phospholipids
PE
CL
AC2PIM2/PI
AC1PIM2
Loading

Supplementary Figure 2: Analysis of apolar and polar phospholipids from GSK3011724A-treated *M. bovis* BCG. (**a**) *M. bovis* BCG cultures were treated with GSK3011724A, TLM and INH and labelled with [^{14}C]-acetate. Drug concentrations are displayed (μM). The total lipid extract was recovered and further solvent partitioned giving rise to apolar and polar phospholipids, which were analysed by autoradiography-TLC (10,000 cpm, equal counts). (**a**) 2D apolar lipid profiles using solvent systems A-C. (**b**) 1D polar phospholipid profile of polar phospholipids. PDIMs, phthiocerol dimycocerosates; TAG, triacylglycerol; MAT, multi-acylated trehaloses; F, fatty acids; GroM, monomycolylglycerol; PGL, phenolic glycolipid; PE, phosphatidylethanolamine; CL, cardiolipin; AC_2PIM_2, diacyl phosphatidylinositol dimannoside; PI, phosphatidylinositol; AC_1PIM_2, acyl phosphatidylinositol dimannoside.

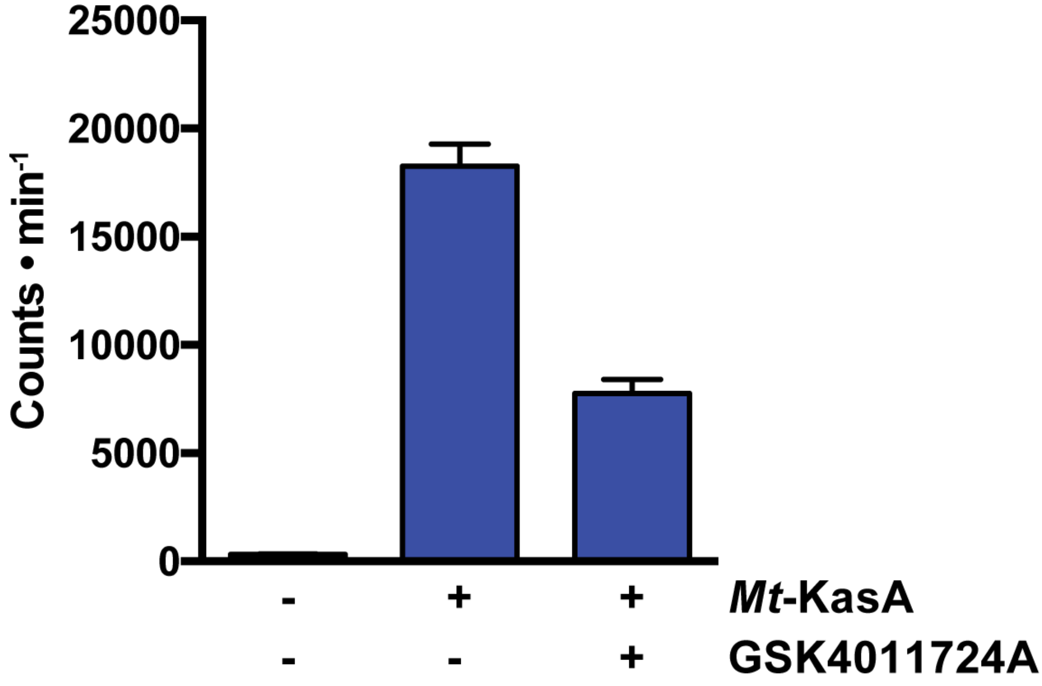

Supplementary Figure 3: Inhibition of KasA-mediated condensation activity by GSK3011724A. KasA activity was monitored by the elongation of C_{16}-AcpM using [^{14}C]-malonyl-AcpM, which had been generated by the translocase reaction of FabD on holo-AcpM and [^{14}C]-malonyl-CoA. The condensation activity of KasA (0.25 μg) was assayed for 1.5 h in 0.1 % (v/v) DMSO with and without GSK3011724A (1 μM). In the absence of KasA, the enzyme was replaced by an equivalent volume of buffer. The results are based on the mean of triplicate reactions, with error bars representing the standard error.

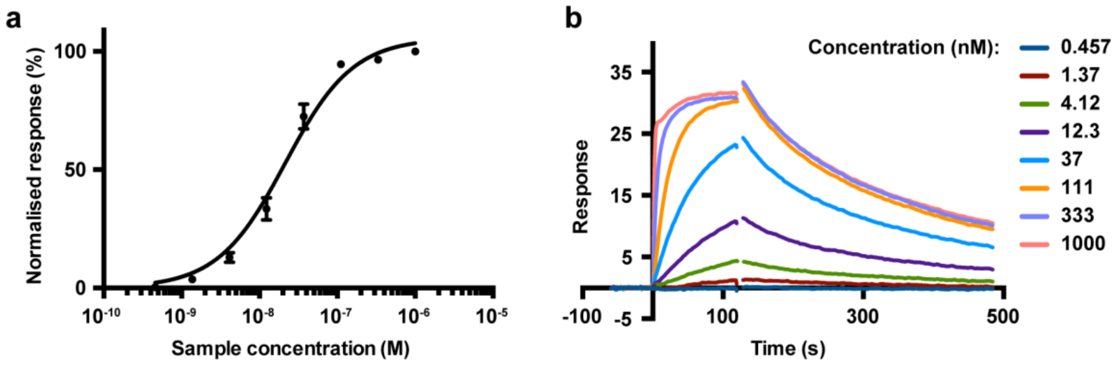

Supplementary Figure 4: Surface Plasmon Resonance binding studies of KasA and GSK3011724A. Purified KasA was immobilised to a chip surface, and the binding of GSK3011724A was monitored. (**a**) Normalised signal from GSK3011724A titrations over immobilised KasA. The results are the mean of triplicate reactions, with error bars representing the standard error. (**b**) Representative sensorgrams of GSK3011724A titrations over immobilised KasA. Kinetic fits are shown.

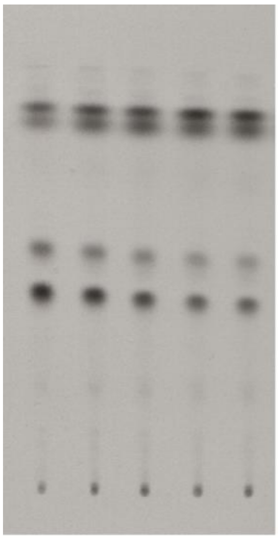

Figure 2a
(left panel)

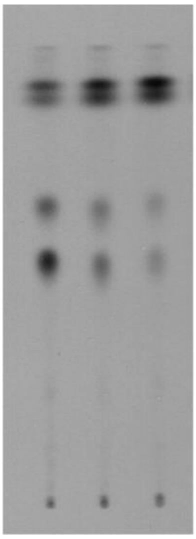

Figure 2a
(middle panel)

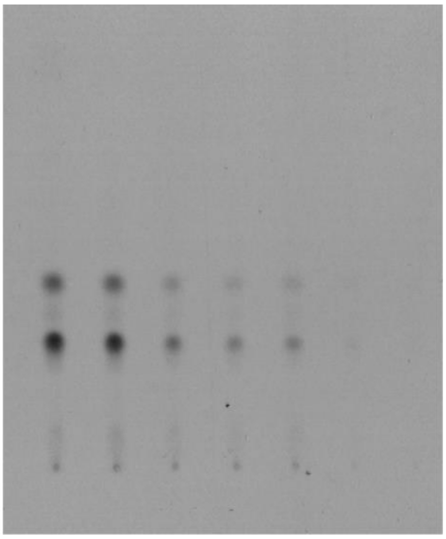

Figure 2a
(right panel)

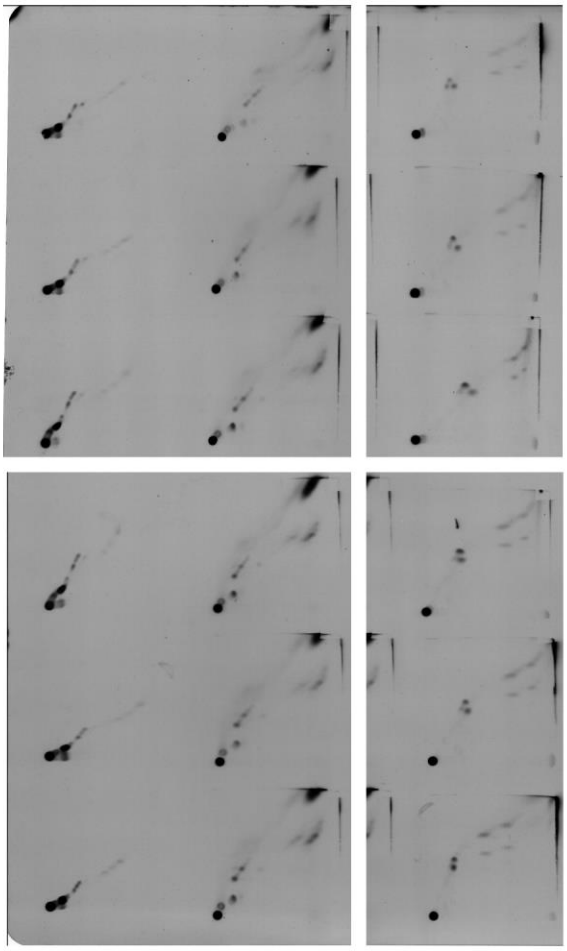

Supplementary
Figure 2a

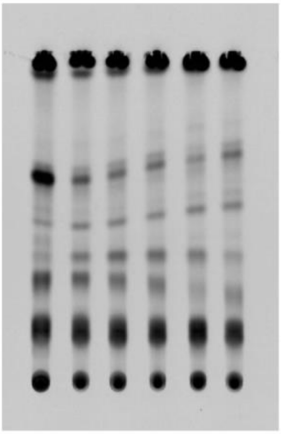

Figure 2b

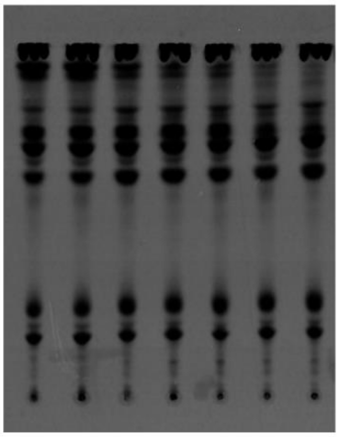

Supplementary
Figure 2b

Supplementary Figure 5: Original scans for all TLC data.

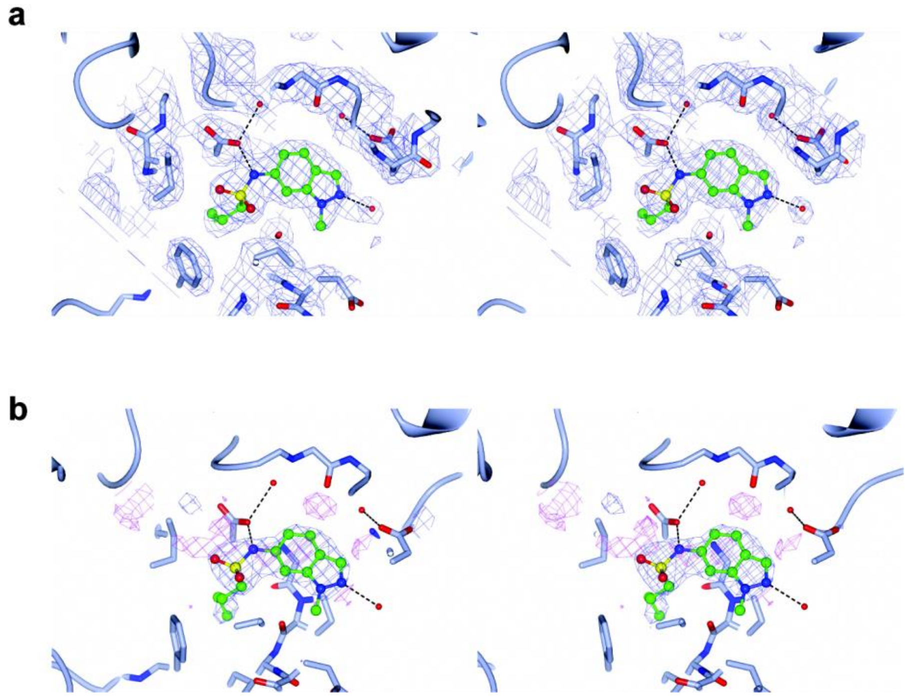

Supplementary Figure 6: Stereo diagram of KasA complexed with GSK3011724A. (**a**) Fo-Fc density map for the ligand (contour level + 1.0 σ (blue)). (**b**) Fo-Fc density map for the ligand (contour level +/- 3.0 σ (blue/pink)).

Supplementary Table

**Assay**	**Mode**	**pXC_{50}**
α1 nicotinic AChR	Opener	<4.3 (2)
α1 nicotinic AChR	Blocker	<4.3 (2)
Acetylcholinesterase	Inhibition	<4 (2)
Adenosine A_{2a}	Agonist	<4 (2)
Adrenergic α_{1B}	Antagonist	<4.6 (2)
Adrenergic α_{2C}	Agonist	<4 (2)
Adrenergic β_2	Agonist	<4 (2)
Adrenergic β_2	Antagonist	<4 (2)
AhR	Agonist	<4 (2)
Aurora B (STK12)	Antagonist	=5.1 (2)
Cannabinoid CB_2	Agonist	<4 (2)
COX-2	Blocker	<4 (2)
Dopamine D_1	Antagonist	<4 (2)
Dopamine D_2	Agonist	<4 (2)
Dopamine D_2	Antagonist	<4 (2)
hERG Ion Works	Antagonist	<4.2 (3)
Histamine H_1	Antagonist	<4.6 (2)
KCNQ1/minK	Blocker	<4.6 (2)
Kv1.5	Blocker	<4.3 (1)
LCK	Antagonist	<4.5 (2)
L-type Ca channel (CaV1.2)	Blocker	<4 (2)
Muscarine M_1	Agonist	<4.3 (2)
Muscarine M_1	Antagonist	<4.3 (2)
Muscarine M_2	Agonist	<4.3 (2)
Muscarine M_2	Antagonist	<4.3 (2)
NaV1.5	Blocker	<4 (2)
Neurokinin NK_1	Antagonist	<4.6 (2)
NMDA Channel (NR2B)	Blocker	<4.3 (2)
Norepinephrine	Antagonist	<4 (2)
OATP1B1	Inhibition	<4.3 (2)
Opioid μ	Agonist	<4 (2)
Opioid κ	Agonist	<4 (2)
PDE_{3A}	Inhibition	<4 (2)
PI3Kγ	Antagonist	<4.5 (1)
PXR	Agonist	<4.3 (2)
Serotonin $5HT_{1B}$	Agonist	<4 (2)
Serotonin $5HT_{1B}$	Antagonist	<4 (2)
Serotonin $5HT_{2A}$	Agonist	<4.6 (2)
Serotonin $5HT_{2A}$	Antagonist	<4.6 (2)
Serotonin $5HT_{2C}$	Agonist	<4.6 (2)
Serotonin $5HT_{2C}$	Antagonist	<4.6 (2)
Serotonin $5HT_3$	Opener	<4.3 (2)
Serotonin $5HT_3$	Blocker	<4.3 (2)
Vasopressin V_{1a}	Antagonist	<4.3 (2)

Supplementary Table 1: Off-target selectivity data for GSK3011724A.

Supplementary Methods

Synthesis and characterisation of compounds

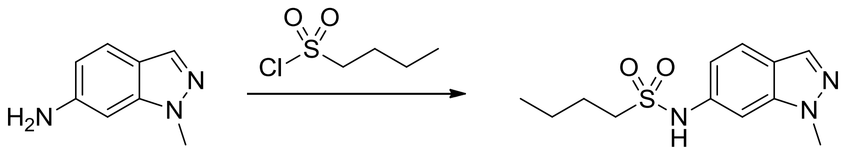

7 **GSK3011724A**

Scheme 1: Synthesis of hit compound GSK3011724A

The synthesis of **GSK3011724A** was performed as follows. Analine **7** (929 mg, 6.31 mmol) was dissolved in dichloromethane (20 mL), and the solution was cooled to 0°C. Triethyl amine (2.64 mL, 18.94 mmol, 3 Eq.) was added followed by the sulfonyl chloride (1.95 mL, 15.78 mmol, 2.5 Eq.) over about 5 min, and then the mixture was allowed to warm to room temperate. After stirring for 3 h, complete consumption of the starting material was observed by LCMS. Methanol (20 mL) and 2 N aqueous sodium hydroxide (12.6 mL, 25.2 mmol, 4 Eq.) were then added to hydrolyse the bis-sulfonamide to the desired mono-sulfonamide, and stirred for 22 h at room temperature. The solution was concentrated to dryness, and the resulting mixture was diluted with dichloromethane and ammonium chloride. The aqueous layer was separated and extracted with dichloromethane, and the combined organic layers were dried over sodium sulfate. The resulting crude mixture was purified first by flash column chromatography (40 g cartridge) eluting with a 0-40 % iPrOH:cHex gradient. After concentration, the resulting coloured solid was re-crystallised from toluene to afford the desired product N-(1-methyl-1H-indazol-6-yl)butane-1-sulfonamide (**GSK3011724A**) as a white solid (1.004 g, 3.57 mmol, 57 %). mp:

120.6–122.2°C. ^{1}H NMR (400 MHz, $CDCl_3$) δ 7.95 (1H, s), 7.68 (1H, d, J = 8.6 Hz), 7.40 (1H, s), 7.15 (1H, s), 7.10 (1H, bs), 6.88 (1H, dt, J = 8.6, 1.5), 4.06 (3H, s), 3.13 (2H, m), 1.83 (2H, m), 1.42 (2H, m), 0.91 (3H, t, J = 7.3 Hz). ^{13}C NMR (100 MHz, DMSO-*d6*) δ ppm: 140.4, 137.3, 132.7, 122.2, 120.7, 115.0, 98.8, 50.6, 35.75, 25.6, 21.1, 13.9. LCMS (ES) [M]+H calculated for $C_{12}H_{17}N_3O_2S$ as 268.1120, found 268.1.

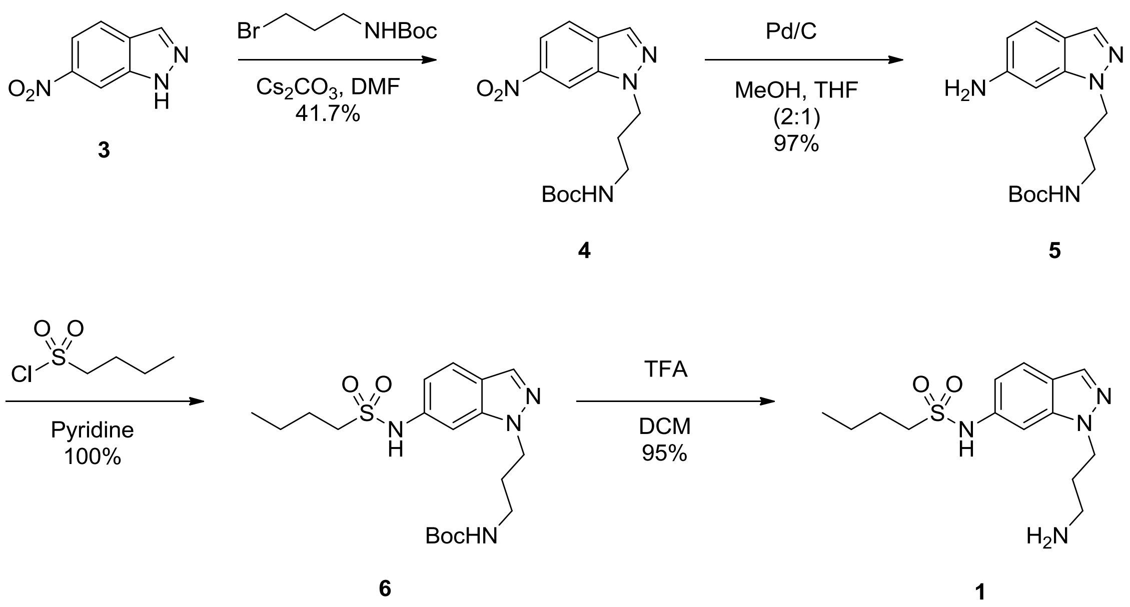

Scheme 2: Synthesis of linkable compound **1**

The synthesis of **1** was performed in a four-step reaction as follows: 6-nitro-1H-indazole (**3**; 203 mg, 1.24 mmol) was dissolved in dry N,N-Dimethylformamide (DMF) (4.1 mL). It was treated successively with caesium carbonate (1216 mg, 3.73 mmol, 3.0 Eq.) and tert-butyl (3-bromopropyl)carbamate (593 mg, 2.49 mmol, 2.0 Eq.). After 16 h the reaction was diluted with ethyl acetate, transferred to a separatory funnel, washed with brine solution 3 x, and the organic layer was dried with anhydrous sodium sulfate, filtered, and concentrated. The crude reaction mixture was purified using a 12 g silica column (30 mL.min^{-1}) on an Isco Brand auto column and was eluted with 40 % EtOAc/Hexanes to afford the desired product, tert-butyl (3-(6-

nitro-1H-indazol-1-yl)propyl)carbamate (**4**) in 41.7 % yield. ^{1}H NMR (400 MHz, $CDCl_3$) δ 8.41 (1H, s), 8.15 (1H, s), 8.05 (1H, d, J = 8.8 Hz), 7.86 (1H, d, J = 8.8 Hz), 4.78 (1H, br s), 4.57 (2H, t), 3.17 (2H, m), 2.19 (2H, m), 1.45 (9H, s); LCMS (ES) [M]+ calculated for $C_{15}H_{20}N_4O_4$ 320.1485, found 321.3 at 0.92 min.

Tert-butyl (3-(6-nitro-1H-indazol-1-yl)propyl)carbamate (58.9 mg, 0.184 mmol) was dissolved in Methanol (1.2 mL) and Tetrahydrofuran (THF) (0.61 mL) and then palladium on carbon (10 %) (11.74 mg, 0.110 mmol) was added. The atmosphere in the reaction flask was removed and replaced by hydrogen gas until the starting material had been consumed (2 h). The crude mixture was filtered through Celite, eluting with EtOAc, dried with anhydrous sodium sulfate and concentrated to afford the desired product, tert-butyl (3-(6-amino-1H-indazol-1-yl)propyl)carbamate (**5**) (51.6 mg, 0.178 mmol, 97 % yield), without further purification. ^{1}H NMR (400 MHz, $CDCl_3$) δ 7.81 (1H, s), 7.49 (1H, d, J = 8.3), 6.57 (1H, d, J = 8.5 Hz), 6.54 (1H, s), 4.85 (1H, br s), 4.32 (2H, t), 3.88 (2H, s br), 3.07 (2H, m), 2.05 (2H, s), 1.43 (9H, s); LCMS (ES) [M]+ calculated for $C_{15}H_{22}N_4O_2$ 290.1743, found 291.3 at 0.53 min.

Tert-butyl (3-(6-amino-1H-indazol-1-yl)propyl)carbamate (51.6 mg, 0.178 mmol) was dissolved in Pyridine (0.854 mL) and was cooled to 0ºC with an ice bath. Butane-1-sulfonyl chloride (35 µL, 0.270 mmol, 1.5 Eq.) was added drop wise. The mixture was stirred at 0°C and then allowed to reach room temperature. After 2 h, the reaction was quenched by addition of water and extracted with EtOAc, dried with $MgSO_4$ and solvent removed. The crude reaction mixture was diluted in EtOAc, washed subsequently with 1.0 N HCl and then brine and the organic solvent was removed *in vacuo*. The crude reaction mixture was dissolved on silica and tert-butyl (3-(6-

(butylsulfonamido)-1H-indazol-1-yl)propyl)carbamate (**6**) was purified using a 4 g silica column (18 mL.min^{-1}) on an Isco Brand auto column, eluted with (30 %) EtOAc/Hexanes. ^{1}H NMR (400 MHz, $CDCl_3$) δ 7.96 (1H, s), 7.68 (1H, d, J = 8.5), 7.42 (1H, s), 7.19 (1H, s), 6.97 (1H, br s), 4.88 (1H, s br), 4.44 (2H, t), 3.12 (4H, m), 2.05 (2H, m), 1.84 (2H, m), 1.44 (11H, m), 0.91 (3H, t); LCMS (ES) [M]+ calculated for $C_{19}H_{30}N_4O_4S$ 410.1988, found 411.4 at 0.83 min.

Tert-butyl (3-(6-(butylsulfonamido)-1H-indazol-1-yl)propyl)carbamate (47.6 mg, 0.116 mmol) was dissolved in DCM (1.739 mL), and Trifluoroacetic acid (TFA) (0.580 mL) was added and stirred for 1 h at room temperature. The reaction was condensed to dryness and the resulting oil was dissolved in MeOH and loaded onto an Isolute SCX column. The material was washed with MeOH and then the desired compound was eluted off of the column using 7.0 N Ammonia in MeOH. The solvent was removed to afford the desired product, N-(1-(3-aminopropyl)-1H-indazol-6-yl)butane-1-sulfonamide (**1**) (34.3 mg, 0.110 mmol, 95 % yield), as an oil. ^{1}H NMR (400 MHz, $CDCl_3$) δ 7.96 (1H, s), 7.67 (1H, d, J = 8.5), 7.45 (1H, s), 6.92 (1H, d, J=8.5), 4.48 (2H, m), 3.26 (3H, s br), 3.13 (2H, m), 2.72 (2H, m), 2.06 (2H, m), 1.83 (2H, m), 1.42 (2H, m), 0.90 (3H, t); LCMS (ES) [M]+ calculated for $C_{14}H_{22}N_4O_2S$ 310.1463, found 311.3 at 0.42 min.

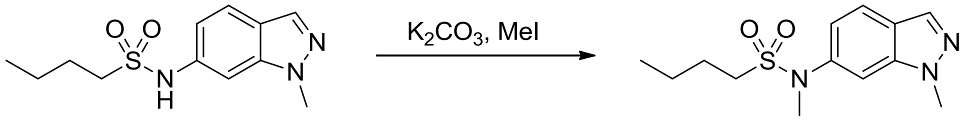

GSK3011724A **2**

Scheme 3: Synthesis of inactive compound **2**

The synthesis of **2** was performed as follows. **GSK3011724A** (110 mg, 0.411 mmol) was dissolved in N,N-dimethylformamide (10 mL), and the solution was cooled to 0°C. Potassium carbonate (171 mg, 1.234 mmol, 3 Eq.) was added followed by iodomethane (100 µL, 1.606 mmol, 4.0 Eq.), and then the mixture was allowed to warm to room temperate and left stirring overnight. The reaction was diluted with water and ethyl acetate. The organic phase was washed several times with aqueous ammonium chloride, dried over sodium sulfate, and concentrated under reduced pressure. The resulting crude mixture was purified first by flash column chromatography (4 g cartridge) eluting with a 0-40 % EtOAc:cHex gradient and concentrated under reduced pressure to afford the desired product N-methyl-N-(1-methyl-1H-indazol-6-yl)butane-1-sulfonamide (**2**) as a white solid (92.5 mg, 0.329 mmol, 80 %). ^{1}H NMR (400 MHz, $CDCl_3$) δ 7.98 (1H, s), 7.73 (1H, d, J = 8.6 Hz), 7.49 (1H, s), 7.15 (1H, s), 7.14 (1H, dd, J = 8.6, 1.8), 4.07 (3H, s), 3.43 (3H, s), 3.02 (2H, m), 1.83 (2H, m), 1.43 (2H, m), 0.93 (3H, t, J = 7.3 Hz). LCMS (ES) [M]+H calculated for $C_{13}H_{19}N_3O_2S$ as 282.1276, found 282.1.

Surface Plasmon Resonance studies

Surface Plasmon Resonance (SPR) binding studies were performed using a Biacore T200 instrument (GE Healthcare) and a NiHC1000m chip (XanTec bioanalytics GmbH, Dusseldorf). Chip preparation and binding analyses were performed at 25°C, with 10 mM HEPES pH 7.4, 150 mM NaCl, 200 µM TCEP, 0.005 % Tween 20, 1 % DMSO as running buffer. After chip conditioning (120 s injection of 0.5 M EDTA, 60 s injection of 500 µM $NiCl_2$, both at 10 $µl.min^{-1}$), KasA was captured by flowing protein (50 $µg.ml^{-1}$ in running buffer) over sample flow-cells for 5-7 min, capturing

10,000 – 15,000 resonance units (RU) of protein. Flow-cell 1 remained unmodified and was used as the reference channel. Binding analyses for GSK3011724A were performed at 30 μl.min^{-1}, using 120 s sample injection times with 1,800 s dissociation. GSK3011724A was diluted to 1 μM in running buffer, then serially diluted 7 times in 3-fold steps, and samples were injected starting with the most dilute. Analyses were repeated on the same surface. Initial dose-responses were discarded due to baseline decay. Only data from matured KasA surfaces, where there was a suitable baseline, were analysed. For each dose-response data set, responses at the end of the association phase were normalised to 0-100%, then combined with replicates and the data fitted with a 1:1 binding model.

***In vitro* death curve assay**

M. tuberculosis H37Rv was cultured at 37°C to OD_{600nm} 0.1-0.8 in Middlebrook 7H9 broth supplemented with Middlebrook ADC and 0.025 % (v/v) tyloxapol. The culture was diluted to 5 x 10^5 CFU.ml^{-1} (10 mL). Cells were treated with 10 x MIC of the following drugs: GSK3011724A (5 μM), moxifloxacin (1.5 μM), linezolid (17.8 μM). A no drug control for positive growth was also included. Cultures were grown in triplicate. A 100 μL aliquot was sampled at 0, 2, 4, 7 and 14 days and the cells were plated onto Middlebrook 7H10 solid media supplemented with Middlebrook OADC. Dilutions corresponding to 10, 100 and 1000 CFU were performed in PBS containing 0.025 % (v/v) tyloxapol, and were plated in triplicate.
